# Drought re-routes soil microbial carbon metabolism towards emission of volatile metabolites in an artificial tropical rainforest

**DOI:** 10.1038/s41564-023-01432-9

**Published:** 2023-07-31

**Authors:** Linnea K. Hernandez, Giovanni Pugliese, Johannes Ingrisch, Jane Fudyma, Juliana Gil-Loaiza, Elizabeth Carpenter, Esther Singer, Gina Hildebrand, Lingling Shi, David W. Hoyt, Rosalie K. Chu, Jason Toyoda, Jordan E. Krechmer, Megan S. Claflin, Christian Ayala-Ortiz, Viviana Freire-Zapata, Eva Y. Pfannerstill, L. Erik Daber, Kathiravan Meeran, Michaela A. Dippold, Jürgen Kreuzwieser, Jonathan Williams, S. Nemiah Ladd, Christiane Werner, Malak M. Tfaily, Laura K. Meredith

**Affiliations:** 1 Biosphere 2, University of Arizona, Tucson, AZ USA; 2 School of Natural Resources and the Environment, University of Arizona, Tucson, AZ USA; 3 Ecosystem Physiology, Faculty of Environment and Natural Resources, University of Freiburg, Freiburg, Germany; 4 Max Planck Institute for Chemistry, Atmospheric Chemistry Department, Mainz, Germany; 5 Department of Ecology, Universität Innsbruck, Innsbruck, Austria; 6 Department of Environmental Sciences, University of Arizona, Tucson, AZ USA; 7 Joint Genome Institute, Walnut Creek, CA USA; 8 Geo-Biosphere Interactions, Department of Geosciences, University of Tuebingen, Tuebingen, Germany; 9 Environmental Molecular Science Laboratory (EMSL), Earth and Biological Sciences Division, Pacific Northwest National Laboratory, Richland, WA USA; 10 Aerodyne Research, Inc., Billerica, MA USA; 11 Department of Environmental Sciences, University of Basel, Basel, Switzerland; 12 Present Address: Department of Plant Pathology, University of California, Davis, CA USA; 13 Present Address: Bruker Daltonics Inc., Billerica, MA USA; 14 Present Address: Department of Environmental Science, Policy, and Management, University of California, Berkeley, CA USA

**Keywords:** Biogeochemistry, Metagenomics, Microbial ecology, Bacterial genes

## Abstract

Drought impacts on microbial activity can alter soil carbon fate and lead to the loss of stored carbon to the atmosphere as CO_2_ and volatile organic compounds (VOCs). Here we examined drought impacts on carbon allocation by soil microbes in the Biosphere 2 artificial tropical rainforest by tracking ^13^C from position-specific ^13^C-pyruvate into CO_2_ and VOCs in parallel with multi-omics. During drought, efflux of ^13^C-enriched acetate, acetone and C_4_H_6_O_2_ (diacetyl) increased. These changes represent increased production and buildup of intermediate metabolites driven by decreased carbon cycling efficiency. Simultaneously,^13^C-CO_2_ efflux decreased, driven by a decrease in microbial activity. However, the microbial carbon allocation to energy gain relative to biosynthesis was unchanged, signifying maintained energy demand for biosynthesis of VOCs and other drought-stress-induced pathways. Overall, while carbon loss to the atmosphere via CO_2_ decreased during drought, carbon loss via efflux of VOCs increased, indicating microbially induced shifts in soil carbon fate.

## Abstract (web summary)

Isotopic labelling and multi-omics experiments conducted in Biosphere 2 show how dry conditions reprogramme microbial metabolism.

## Main

Microorganisms regulate terrestrial carbon (C) cycling in fundamental ways^1^, including transforming soil C into gaseous compounds that can escape to the atmosphere, primarily as CO_2_ via microbial heterotrophic respiration. However, microbes also produce volatile organic compounds (VOCs) as metabolic intermediates, signalling molecules and secondary metabolites^2,3^. In fact, volatile metabolites represent an often overlooked subset of the complete soil metabolome^4,5^, and although their emissions to the atmosphere only represent a small soil C loss, they contribute substantially to atmospheric chemistry including ozone formation and cloud condensation nuclei^6^. Therefore, characterizing microbe-mediated C flow along the soil–atmosphere continuum is critical for understanding the fate of soil C and VOCs under projected environmental changes, including drought.

Drought stress induces well-characterized microbial physiological responses that impact C metabolism, such as biosynthesis of protective molecules (for example, osmolytes and extracellular polymeric substances) to preserve cell integrity^7,8^ and concentrate resources^9,10^. Production of these biomolecules is energy intensive and may divert resources from biomass synthesis^9^, leading to decreased growth-fuelling heterotrophic respiration and CO_2_ emissions^11,12^. Drought also induces changes in soil C composition and availability^13–15^, further impacting microbial activity^16,17^, for example, by inducing shifts in substrate utilization^18^. Overall, it remains unclear how drought-induced shifts in microbial metabolism and soil C composition influence C allocation to volatile metabolites, which can mitigate drought stress in plants^19^. Moreover, soil water content has a strong impact on VOC emissions from soils^20^, including tropical soils^21,22^, perhaps driven by drought impacts on microbial VOC biosynthesis and/or consumption. Characterizing changes in microbial C cycling and allocation is particularly important in tropical rainforest soils where droughts will probably occur more frequently and last longer due to climate change^23,24^.

Detecting shifts in microbial C cycling and allocation within complex metabolic networks encompassing competing production and consumption pathways is challenging. This complexity can be navigated by tracking microbe-mediated C flow through soils using isotopically labelled central metabolites. Position-specific ^13^C-glucose and/or ^13^C-pyruvate labelling has been used to track microbial C allocation to CO_2_ and biomass in soil mesocosms^25–27^. However, studies on drought in the field and allocation to VOCs are missing^28^. The direct metabolic information derived from isotope labelling can be contextualized using powerful, information-rich constraints provided by ‘omics approaches that profile the gene content, gene expression and metabolomes of soil microbiomes^26^. Together, these approaches can uncover metabolic drivers of shifting microbial C cycling and allocation in soils under drought.

In this study, we performed a comprehensive assessment of how drought impacts soil microbial C allocation to CO_2_ and VOCs in an artificial enclosed tropical rainforest at Biosphere 2 using an integrated approach, combining position-specific ^13^C-pyruvate labelling with metatranscriptomics, metagenomics and metabolomics. Biosphere 2 (Oracle, Arizona) is a 12,700 m^2^ steel and glass-enclosed building harbouring five distinct biomes, including a 1,900 m^2^ tropical rainforest—an enclosed ecosystem with variable topography and microhabitats where rain and temperature can be controlled. In 2019, a 65-day drought was imposed on the tropical rainforest to study ecosystem-scale impacts of dry-down conditions^29^. With the assumption that the C1 position of pyruvate would enter CO_2_ via decarboxylation during biosynthesis or respiration (tricarboxylic acid (TCA) cycle), and the C2 position would enter VOCs (as biosynthesis) or CO_2_ during respiration^26,27^, we aimed to infer microbial C allocation to biosynthesis vs TCA cycle during both ambient and drought conditions. We hypothesized that during drought: (1) microbial C allocation to the TCA cycle would decrease as C allocation is diverted to increased biosynthesis of stress compounds, including VOCs and (2) changes in C allocation would be associated with shifts in metabolic composition and gene expression.

## Results

### Gaseous emissions from soil

**Fig. 1:**
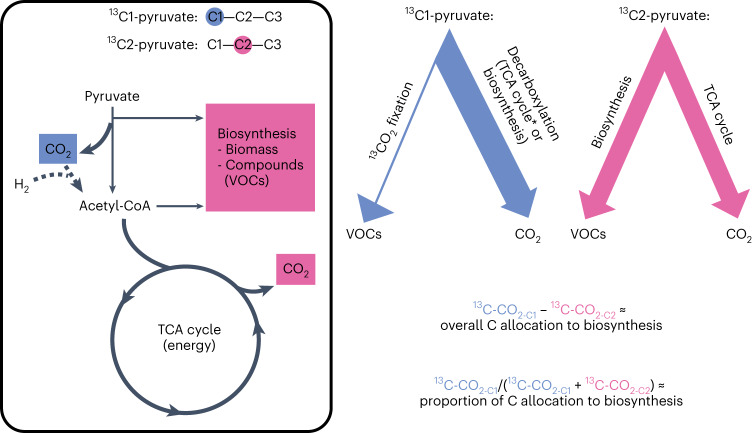
C allocation from pyruvate into CO_2_ and VOCs. Framework depicting how first (C1; blue) and second (C2; pink) carbon (C) positions of pyruvate from ^13^C1-pyruvate and ^13^C2-pyruvate, respectively, are expected to be allocated into CO_2_ and biosynthetic (including VOCs) pathways. As indicated by the arrow thickness, we expect most C1 to be released as CO_2_ during biosynthesis and TCA cycle, and C2 to be either released as CO_2_ in the TCA cycle or used for biosynthesis of biomass and products. The difference in ^13^C-CO_2-C1_ − ^13^C-CO_2-C2_ approximates the total C allocation to biosynthesis, while the ratio ^13^C-CO_2-C1_ / (^13^C-CO_2-C1_ + ^13^C-CO_2-C2_) approximates the proportion of internal C allocation to biosynthesis. *Some ^13^C from ^13^C1-pyruvate may end up in the TCA cycle due to anaplerotic CO_2_ assimilation, which would lead to a slight overestimate in the proportion of C allocation to biosynthesis and overall C allocation to biosynthesis. Figure adapted with permission from refs. ^26,27^, Elsevier.

**Fig. 2:**
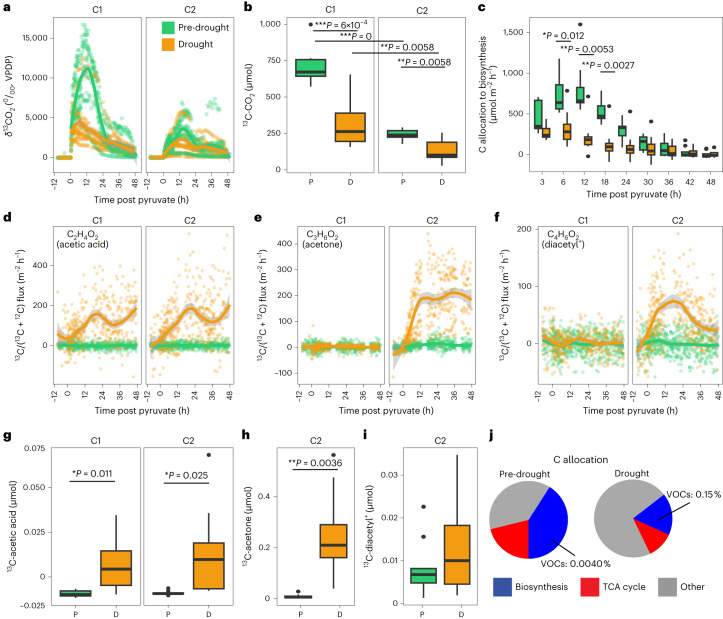
Drought induces a shift in C allocation, as measured using position-specific ^13^C-pyruvate labelling. **a**, *δ*^13^C of CO_2_ efflux over time from 12 h pre to 48 h post pyruvate injection from both ^13^C1- and ^13^C2-pyruvate. **b**, Cumulative ^13^C-CO_2_ soil efflux from 0 to 48 h post pyruvate injection. **c**, Overall C allocation to biosynthesis (calculated as ^13^C-CO_2-C1_ − ^13^C-CO_2-C2_). Continuous emission data were binned to 3 or 6 h intervals. **d**–**f**, Fluxes of ^13^C-enriched (^13^C/(^12^C + ^13^C)) acetic acid (**d**), acetone (**e**) and C_4_H_6_O_2_ (diacetyl^+^) (**f**) from 0 to 48 h post pyruvate injection. **g**–**i**, Cumulative flux of ^13^C-acetic acid (**g**), ^13^C-acetone (**h**) and ^13^C-diacetyl^+^ (**i**) ^13^C-diacetyl+ from chambers that received ^13^C1- and/or ^13^C2-pyruvate, if ^13^C-enrichment was evident (see panels **d**–**f**). **j**, Pie charts of the percentage of ^13^C from pyruvate allocated to biosynthesis or the TCA cycle (or other which could represent unmetabolized pyruvate, CO_2_ fixation or uncharacterized pathways) for pre-drought (P) and drought (D). For all time series (**a**,**d**–**f**), each point represents a single measurement per soil chamber (~64 measurements), of which there were 3 replicates each that were injected with either ^13^C1- or ^13^C2-pyruvate for each site (total *n* = 18 each for pre-drought and drought), and lines show the smoothed data with the surrounding shaded area showing ±s.e.m. Boxplots represent Q1–Q3, centre lines indicate the median and whiskers extend to the minimum and maximum values, exclusive of outliers (black points), of cumulative flux (measured per soil chamber) across all sites and timepoints (**b**,**g**–**i**) (*n* = 54 each for pre-drought and drought) or overall C allocation to biosynthesis (measured per set of C1/C2 chambers) across sites and specific timepoints as indicated (**c**) (*n* = 9 sets each for pre-drought and drought). *P* values in **b**, **c**, **g**–**i** are based on linear mixed dffects models. *P < 0.05, **P < 0.01, ***P < 0.001. Source data

**Extended Data Fig. 1:**
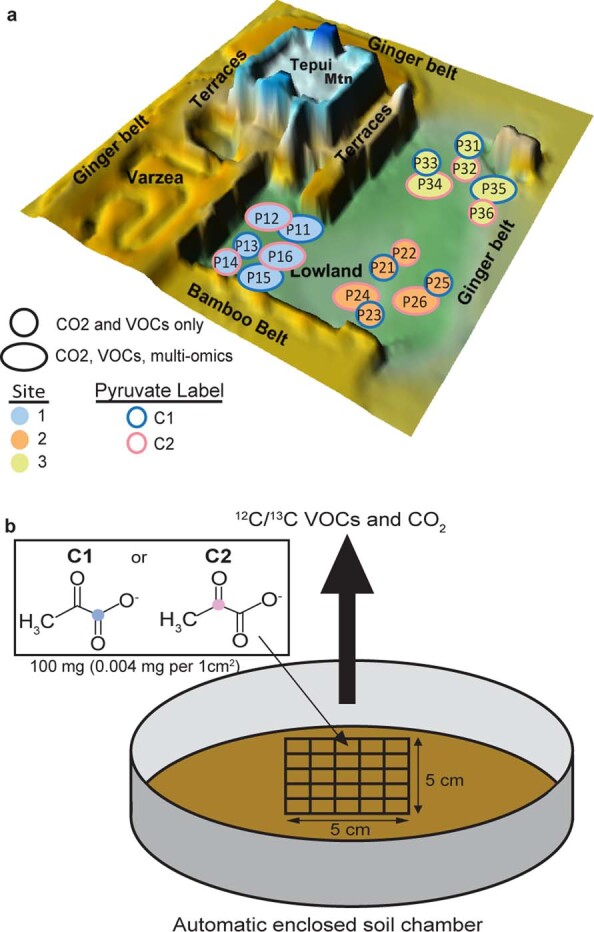
Soil chamber locations and experimental setup. **a)** Topological map of Biosphere 2 Tropical Rainforest, with locations of Sites 1 − 3. All circles represent collar locations where CO_2_ and VOC data were collected and large circles represent a sub selection of sites collars where additional soil samples were collected for metatranscriptomics, metagenomics, and metabolomics. **b)** Diagram showing soil pyruvate experimental setup with stencil placed inside a soil collar where ^13^C1- or ^13^C2-pyruvate was added within each 1 cm×1 cm square. An automatic soil chamber was placed over the soil collar during the experiment and continuous measurements of ^12^C and ^13^C-CO_2_ and VOCs were measured.

**Extended Data Fig. 2:**
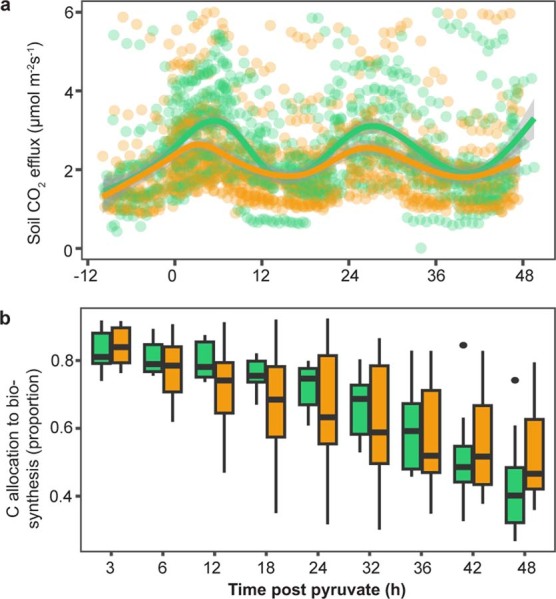
Continuous CO_2_ emissions and proportion of C from pyruvate allocated to biosynthesis. **a)** Total flux of CO_2_ during pre-drought and drought conditions. Each point represents a single measurement per soil chamber (~ 64 measurements), of which there were 3 replicates each that were injected with either ^13^C1- or ^13^C2-pyruvate for each site (total n = 18 each for pre-drought and drought), and lines show the data smoothed with the surrounding shaded area showing ± SEM. B) Proportion of C from pyruvate allocated to biosynthesis. This was calculated as ^13^C-CO_2-C1_/ (^13^C-CO_2-C1_ + ^13^C-CO_2-C2_) on each set of C1/C2 chambers per site (n = 9 each for pre-drought and drought) with continuous emission data binned to 3 or 6 h intervals, from 0 to 48 h post pyruvate injection. (n = 9 each for pre-drought and drought). Boxes represent Q1 - Q3 with center line indicating median, and bars extending to maximum and minimum values, excluding outliers. Source data

We tracked ^13^C from the first and second positions of pyruvate (^13^C1-pyruvate and ^13^C2-pyruvate, respectively) into CO_2_ and VOCs (Fig. 1) within soil chambers located among three sites across the tropical rainforest at Biosphere 2 (Extended Data Fig. 1). During drought, total soil CO_2_ efflux and its ^13^C-enrichment (*δ*^13^C_CO2_) decreased after injection of ^13^C1-pyruvate and ^13^C2-pyruvate relative to pre-drought, with higher *δ*^13^C_CO2_ from ^13^C1-pyruvate compared with ^13^C2-pyruvate chambers during both pre-drought and drought (Fig. 2a and Extended Data Fig. 2a). As expected, this pattern was reflected in the cumulative ^13^C-CO_2_ effluxes (Fig. 2b), which decreased significantly during drought by 54.3% from chambers receiving ^13^C1-pyruvate (*t*-value = −5.85, d.f. = 7, *P* = 0, linear mixed effect (LME)) and 47.6% from chambers receiving ^13^C2-pyruvate (*t*-value = −3.92, d.f. = 7, *P* = 0.0058, LME).

Contrary to our hypothesis, microbes maintained their allocation to energy production during drought-induced stress. While overall C allocation to biosynthesis was higher during pre-drought conditions between 3 and 18 h post pyruvate addition (Fig. 2c), internal partitioning of C to biosynthesis vs TCA cycle did not change (Extended Data Fig. 2b).

**Extended Data Fig. 3:**
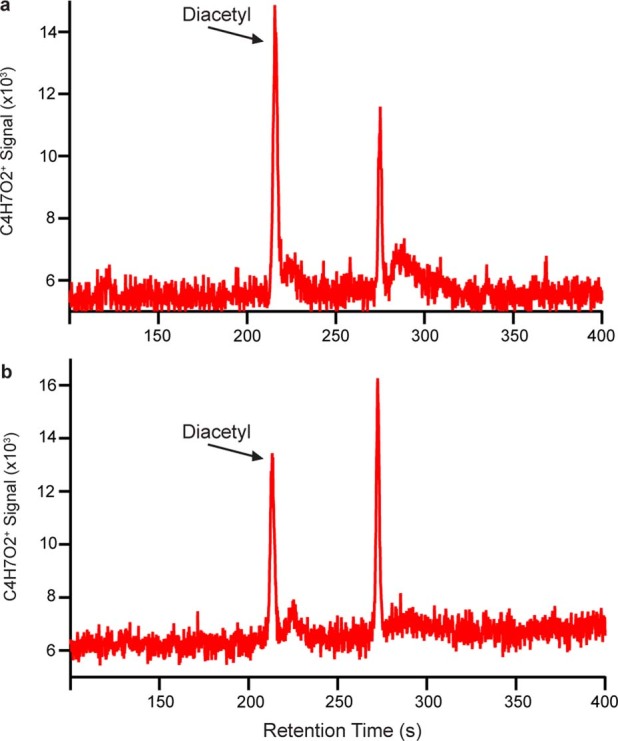
GC-PTR-TOF data to identify C_4_H_6_O_2_. Two nearby locations **a)** and **b)** had two peaks with retention times 217 s and 275 s for C_4_H_6_O_2_ corresponding to two different compounds. The retention time of 217 s matched the expected retention time for diacetyl, but the retention time of 275 s did not match with any known compounds. Therefore, C_4_H_6_O_2_ may represent diacetyl and/or one additional unidentifiable compound, and is referred to as diacetyl^+^ in the manuscript. The diacetyl peak (retention time of 217 s) is indicated with an arrow.

To further reveal C allocation strategies, we tracked ^13^C from pyruvate into ^13^C-enriched (^13^C/(^12^C + ^13^C)) volatile compounds and were only able to detect three, all of which are central metabolites: acetone, acetic acid and C_4_H_6_O_2_ (Fig. 2d–f). Cumulative effluxes of ^13^C-acetic acid and ^13^C-acetone increased significantly by factors of 2.6 and 25.3, respectively, during drought from chambers receiving ^13^C2-pyruvate (*t*-values = 2.83 and 4.30, respectively; d.f. = 7, *P* < 0.05, LME) (Fig. 2g,h), indicating synthesis along metabolic pathways after C1 decarboxylation to CO_2_. Emissions of acetone also increased during low moisture in a previous study in the tropical rainforest^21^, and these results suggest a role of microbes in the production of acetone and consequent emissions during drought. Acetic acid also showed ^13^C-enriched continuous efflux (Fig. 2e,f) from chambers that received ^13^C1-pyruvate where ^13^C-acetic acid cumulative efflux increased significantly by a factor of 1.9 during drought (*t*-value = 3.45; DF = 7; *P* < 0.05; LME) (Fig. 2g). ^13^C-enrichment of acetic acid from chambers receiving ^13^C1-pyruvate indicates active acetogenesis, where acetogens fix two CO_2_, in this case ^13^C-CO_2_, and four H_2_ molecules to form acetyl-CoA, which is further reduced to acetate^30^ (Fig. 1), an intermediate of central metabolism that can be protonated in soils and volatilized as acetic acid (hereafter referred to as acetate). C_4_H_6_O_2_ probably comprised diacetyl and/or one other unidentifiable compound (henceforth referred to as diacetyl^+^) based on structural identification of compounds with this formula from nearby locations (Extended Data Fig. 3). ^13^C may have been allocated to other non-volatile metabolites; however, no such ^13^C-enrichment was detected by ^1^H-nuclear magnetic resonance (NMR).

Drought induced a readjustment of overall C allocation. The proportion of ^13^C from pyruvate allocated to biosynthesis dropped from 41.0 to 17.3% and to the TCA cycle from 21.1 to 11.1% during drought (Fig. 2j). Despite this overall decrease in C allocation to biosynthesis, ^13^C-enriched emissions of acetate, acetone and diacetyl^+^ represented a greater proportion of biosynthetic products during drought, increasing from 0.0040% to 0.15% (Fig. 2j). Meanwhile, ^13^C from pyruvate that did not track to emitted ^13^C-CO_2_ (‘other’), possibly representing unmetabolized pyruvate and/or autotrophic and heterotrophic CO_2_ fixation, increased from 37.9 to 71.6% during drought (Fig. 2j). Total CO_2_ fixation would be expected to decrease CO_2_ emissions by up to 5.6%, depending on soil type^30,31^, suggesting the potential for only a small impact on our estimates of microbial C allocation to biosynthesis vs TCA cycle. Overall, these data suggest an increase in biosynthesis of VOCs during drought, despite an overall decrease in microbial C allocation to biosynthesis.

### Acetate, acetone and diacetyl^+^ cycling gene expression

**Fig. 3:**
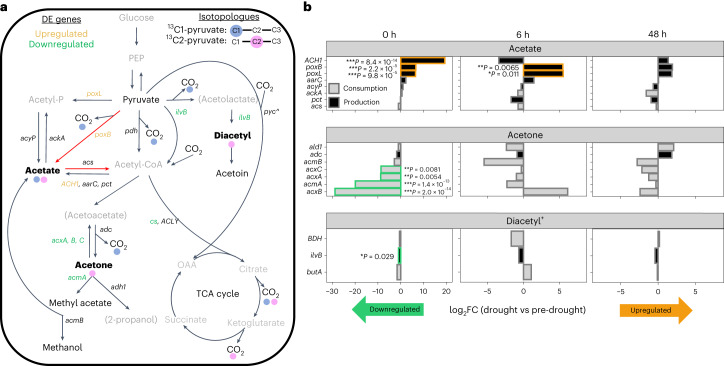
Expression of genes encoding enzymes that cycle ^13^C-enriched VOCs shifts with drought. **a**, Pyruvate metabolism pathways that lead to ^13^C-enriched VOCs and CO_2_, shown with blue and pink circles, to represent incorporation of ^13^C-pyruvate from the C1 and/or C2 position, respectively. Colour code for compound names: grey names without brackets indicate compounds that were not measured, grey names with brackets indicate VOCs that were not emitted, plain black names indicate VOC fluxes that were emitted but not ^13^C-enriched and bold black names indicate VOC emissions that were ^13^C-enriched (acetate, acetone, diacetyl^+^). Red arrows represent a potential PDH bypass route of acetyl-CoA production from pyruvate to acetate to acetyl-CoA. Green and orange gene names signify down- or upregulation during drought, respectively, compared to pre-drought conditions. **b**, Changes in gene expression across all sites (for pre-drought: 0 and 48 h (*n* = 6), 6 h (*n* = 5); for drought: *n* = 6 for each timepoint) as log_2_ fold-change (log_2_FC) of drought vs pre-drought for acetate, acetone and diacetyl^+^ (C_4_H_6_O_2_) at 0, 6 and 48 h post pyruvate addition. Genes that encode enzymes involved in production are in black bars and consumption in grey bars. *P* values in **b** are based on DESeq2 and are FDR corrected. ^, anaplerotic CO_2_ assimilation via *pyc* (pyruvate carboxylase) leading to some ^13^C from ^13^C1-pyruvate entering the TCA cycle and being released as ^13^C-CO_2_. *acxA–C*, acetone carboxylase subunits A–C; *ilvB*, acetolactate synthase; *cs*, citrate synthase; *ald1*, NAD^+^-dependent secondary alcohol dehydrogenase; OAA, oxaloacetate; PEP, phosphoenolpyruvate. *P < 0.05, **P < 0.01, ***P < 0.001. Source data

**Extended Data Fig. 4:**
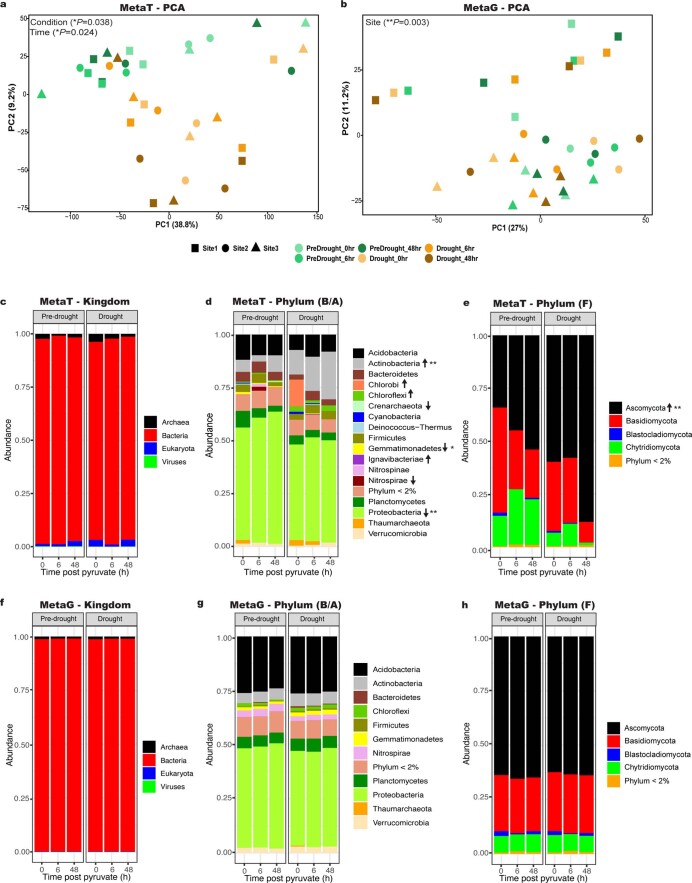
Metatranscriptomic and metagenomic functional and taxonomic diversity shows shifts in active microbial functional profiles and taxonomic diversity, but not in functional potential and total (active and inactive) taxonomic diversity. PCA of **(a)** metatranscriptomic and **(b)** metagenomics data. Taxonomic profiles as inferred from metatranscriptomics data at the **(c)** kingdom-level for active archaea, bacteria, eukaryota, and viruses, **d)** phylum-level for bacteria and archaea, and **e)** phylum-level for fungi. Taxonomic profiles as inferred from metagenomics data at the **f)** kingdom-level for total archaea, bacteria, eukaryota, and viruses, **g)** phylum-level for bacteria and archaea, and **h)** phylum-level for fungi. For taxonomic profiles, arrows indicate direction of changes in relative abundance (up for increased, and down for decreased relative abundance in drought compared to pre-drought conditions). Arrows with no stars means the phylum was present in one condition (pre-drought or drought) and absent in the other condition. For panels **a**-**b**, P-values were determined using PERMANOVA on Bray-Curtis distance matrices. For panels **c**-**h**, *P*-values were determined using Linear Mixed Effect Models. *, *P* < 0.05; **, *P* < 0.01; (exact P-values: *P* = 0.010 [Proteobacteria], *P* = 0.016 [Gemmatimonadetes], *P* = 0.0012 [Actinobacteria] (panel **b**), *P* = 0.0050 [Ascomycota] (panel **c**)). metaT, metatranscriptomics; metaG, metagenomics; B, bacteria; A, archaea; F, fungi. Source data

To characterize active microbial metabolic pathways that could cycle acetate, acetone and diacetyl^+^ in soils, we utilized a gene-centric approach using metatranscriptomics and metagenomics data to identify: (1) specific genes that may be driving the ^13^C-enriched VOC emissions (see Fig. 3a for possible metabolic pathways leading from pyruvate to acetate, acetone and diacetyl, and Extended Data Fig. 6 for associated gene expression (acetate and acetone only)) and (2) overall gene expression patterns that reflect ecosystem-scale microbial responses to drought. Drought and ^13^C-pyruvate injection (that is, comparison between 0 (before injection), 6 and 48 h post-injection timepoints) significantly affected overall gene expression profiles (*P* < 0.05, permutational multivariate analysis of variance (PERMANOVA)) (Extended Data Fig. 4a) and taxonomic composition of active microbial communities (Extended Data Fig. 4c–e) but had no impact on microbial function potential (Extended Data Fig. 4b) or taxonomic composition (Extended Data Fig. 4f–h). Only location (site) impacted microbial functional potential (*P* = 0.003, PERMANOVA) (Extended Data Fig. 4b). This demonstrates that fluctuating gene expression was driven by changes in microbial activity and not by drastic shifts in community composition. We point out the caveat that these results are based on known genes and pathways within the Kyoto Encylopedia of Genes and Genomes (KEGG) database, potentially leading to bias against some organisms that are underrepresented in the databases, such as protists^32^.

Both ^13^C-enriched acetate and acetone effluxes from chambers that received ^13^C2-pyruvate (acetate-C2 and acetone-C2, respectively) were driven by a combination of genes encoding for production and consumption, as well as soil moisture, which together explained 91% of acetate-C2 and 76% of acetone-C2 efflux (partial least square regression (PLSR)) (Table 1). Soil moisture was a major driver of both acetate-C2 and acetone-C2 effluxes (VIP = 1.02 and 1.00, w1 = −0.42 and −0.44; PLSR) (Table 1), indicating that emission of these microbially produced VOCs was partly driven by drought-induced moisture changes. Previous research found that decreased soil moisture is associated with increased movement of volatile compounds through the soil^33^.

**Table 1:** PLSR model summaries of ^13^C-enriched VOCs (acetate, acetone and diacetyl^+^) fluxes after labelling with ^13^C2-pyruvate

VOC model	Gene	KO	Produce (p) or consume (c)	Variable (enzyme/VOC efflux/physiochemical)	VIP	PLSR weight	
						w1	w2
Acetate	na	na	na	acetone-C2 efflux	1.51	0.61	0.28
	*ACH1*	K01067	p	acetyl-CoA hydrolase	1.21	0.48	0.15
	*aarC*	K18118	c	succinyl-CoA:acetate CoA-transferase	1.05	0.21	−0.23
	na	na	na	soil moisture (%)	1.02	−0.42	–
	*poxL*	K00158	p	pyruvate oxidase	0.92	0.17	−0.53
	*poxB*	K00156	p	PDH-quinone	0.90	0.18	−0.50
	*acyP*	K01512	p	acylphosphatase	0.86	–	0.28
	*acs*	K01895	c	acetyl-CoA synthase	0.82	−0.21	0.12
	*pct*	K01026	p	propionate CoA-transferase	0.82	−0.28	–
	*ackA*	K00925	c	acetate kinase	0.75	–	0.46
				***R***^**2**^***-*** **total 0.91 (6 comp.)**		**0.51**	**0.20**
Acetone	na	na	na	acetate-C2 efflux	1.36	0.61	–
	*adc*	K01574	p	acetoacetate decarboxylase	1.05	0.47	–
	na	na	na	soil moisture (%)	1.00	−0.44	–
	*adh1*	K18382	c	NAD^+^-dependent secondary alcohol dehydrogenase	0.80	0.36	–
	*acmA*	K18371	c	acetone monooxygenase	0.64	−0.29	–
				***R***^***2***^***-*** **total 0.76 (1 comp.)**		**0.76**	***–***
Diacetyl^+^	na	na	na	soil moisture (%)	1.12	–	0.99
	*butA*	K03366	c	meso-butanediol dehydrogenase	0.86	–0.99	–
				***R***^**2**^***-*** **total 0.23 (2 comp.)**		**0.15**	**0.08**

PLSR models were created with single response variables (fluxes for each of the ^13^C-enriched VOCs) and predictor variables that included expression of genes known to produce or consume each ^13^C-enriched VOC and soil moisture (%). The VIP values represent the degree of importance of each predictor variable on the response variable. The PLSR weights (w) depict the magnitude and direction of contribution of each predictor variable (+ or − relationship) on the response variable along the first and/or second component (w1 and w2), with a dash indicating a lack of contribution to that component. Genes or physicochemical properties that were determined to not contribute to each model are not shown (see Methods). ^13^C-enriched VOC fluxes and gene expression data are from three collars that received ^13^C2-pyruvate at 0, 6 and 48 h post pyruvate injection during pre-drought and drought conditions (*n* = 18). The total percentage of variance explained (*R*^2^) from predictor variables for all components (comp., shown in parentheses) in the optimized model is shown in the bottom row for each VOC model in bold. The *R*^2^ broken down by the first and second components are shown below w1 and w2, respectively, in bold. Acetone-C2 efflux was included in the model for acetate-C2, and acetate-C2 efflux was included in the model for acetone-C2 due to the high correlation between acetate-C2 and acetone-C2.

na, not applicable; +, C_4_H_6_O_2_ represents diacetyl and/or one other unidentifiable compound.

**Extended Data Fig. 5:**
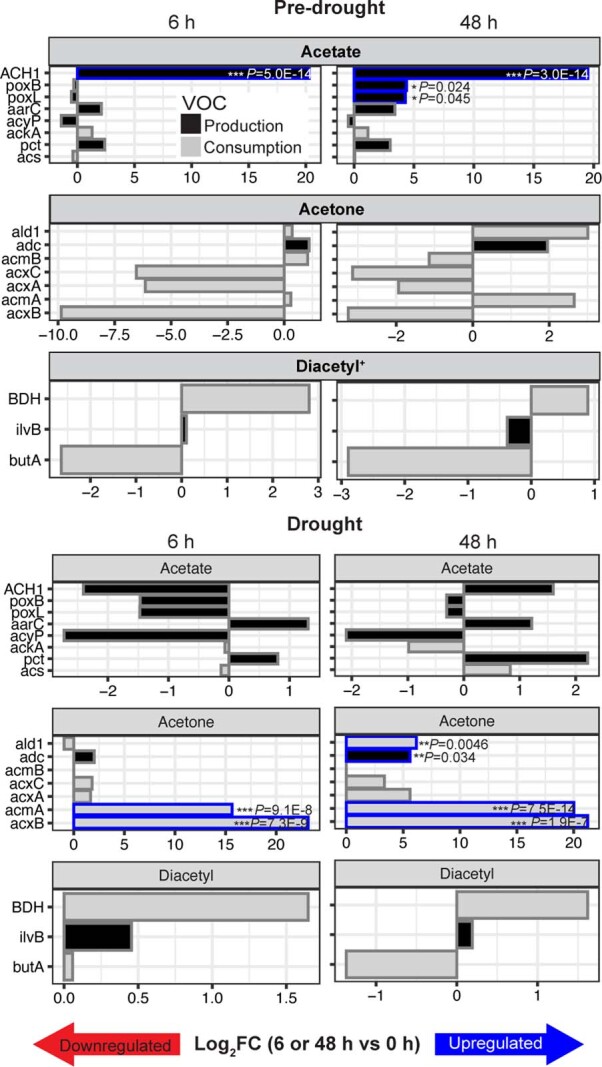
Up- or down-regulation of genes encoding for enzymes that cycle ^13^C-enriched VOCs shift with pyruvate addition. Log_2_-fold change (FC) of genes involved in cycling of acetate, acetone, and diacetyl^+^ at 6 and 48 h in relation to 0 h to show effect of pyruvate addition on microbial activity during **a)** pre-drought and **b)** drought conditions. Log_2_-FC values to indicate up-or down-regulation calculated with DESeq2. Production vs. consumption indicated by black and grey bars, respectively. Significantly up- or down-regulated genes are outlined in color (blue = upregulated, red = downregulated), with exact *P*-values from DESeq2 analysis shown.); *, *P* < 0.05; **, *P* < 0.01; ***, *P* < 0.001. Source data

Microbial gene expression that drove acetate-C2 efflux primarily included acetate-producing enzymes, three of which were upregulated during drought (Fig. 3b). Expression of *ACH1* (K01067), encoding for an acetate-producing acetyl-CoA hydrolase which was upregulated during drought (*P* = 8.4 × 10^−14^, differential analysis using DESeq2) (Fig. 3b and Extended Data Fig. 5b), was the largest microbial driver of acetate-C2 efflux (VIP = 1.51; w1 = 0.51; PLSR) (Table 1). *ACH1*, only present in eukaryotes^33–35^, including fungi^33,35^, produces acetate and CoA from acetyl-CoA^36^, and can play a role in acetyl-CoA regulation. The two other largest microbial drivers of acetate-C2 efflux were expression of *poxB* (K00156; encoding pyruvate dehydrogenase (PDH)-quinone) and *poxL* (K00158; encoding pyruvate oxidase) (VIP = 0.90 and 0.92, w1 = 0.17 and 0.18, respectively; PLSR) (Table 1). The *poxB* gene, first identified in *Escherichia coli*^37,38^, encodes for the protein PDH-quinone, which catalyses the direct conversion of pyruvate to acetate and CO_2_ via oxidative decarboxylation, with quinone or menaquinone as the electron acceptor^37,38^. Quinone then shuttles the electrons to the electron transport chain where O_2_ is the final electron acceptor, thereby producing ATP^39^. The strong upregulation of *poxB* during drought suggests that this gene may improve microbial fitness during drought, perhaps to gain extra energy for biomolecule production, as discussed below.

Microbial gene expression that drove acetone-C2 efflux was limited to three genes: two encoding for acetone-consuming enzymes and one for acetone production. Expression of *adc* (K01574), encoding for the acetone-producing enzyme acetoacetate decarboxylase, was the largest microbial driver of acetone-C2 efflux (VIP = 1.05, w1 = 0.61; PLSR) (Table 1); however, *adc* expression did not change during drought (Fig. 3b). The gene *adc* is part of the acetone-butanol-ethanol (ABE) fermentation pathways in *Clostridium acetybuticulum* and related species^40,41^; however, we did not see the expected concurrent ^13^C-enriched ethanol or butanol emissions, indicating either the presence of other routes of acetone production, or maintained consumption of ethanol and butanol under drought conditions. The other two genes that drove acetone-C2 efflux were *adh1* (K18382) and *acmA* (K18371), which encode for the acetone-consuming enzymes alcohol dehydrogenase (producing propanol, which was not detectable due to its extreme fragmentation during ionization with proton-transfer-reaction mass spectrometry (PTR–MS))^42^ and acetone monooxygenase (producing methyl acetate), respectively (VIP = 0.84 and 0.64, respectively; PLSR) (Table 1). *AcmA* was also downregulated during drought, along with three other genes encoding for acetone-consuming enzymes (*acxA* (K10855), *acxB* (K10854) and *acxC* (K10586)) (*P* < 0.05; DESeq2) (Fig. 3b). While C_3_H_6_O_2_ was detected, possibly indicating methyl acetate, it was not ^13^C-enriched. It is possible that methyl acetate was further broken down into methanol and ^13^C-acetate via methyl acetate hydrolase (*acm*; K18372); however, *acm* was not a major driver of acetone-C2 or acetate-C2 efflux. Therefore, the overall significant decrease in acetone-C2 efflux was driven by both increased production and decreased consumption during drought, signifying that changes in VOC fluxes from soils depend not only on production but also on internal consumption.

For diacetyl^+^-C2 efflux, gene expression and soil moisture accounted for only 23% of total variation (Table 1). Diacetyl is formed when *ilvB* (K01652), encoding for the enzyme acetolactate synthase, catalyses the conversion of pyruvate to CO_2_ and acetolactate, which is further converted non-enzymatically to diacetyl^43^. During drought, *ilvB* was downregulated (*P* = 0.029; DESeq2) (Fig. 3b) and its expression was not a major driver of diacetyl^+^-C2 efflux. Only the expression of *butA* (K03366) was a microbial driver of diacetyl^+^-C2 efflux (VIP = 1.05; PLSR) (Table 1). *ButA* encodes for meso-butanediol dehydrogenase, an enzyme that plays a diacetyl-consuming role producing acetoin (Fig. 3a), although there was no significant change in expression of *butA* during drought (Fig. 3b). The unidentifiable C_4_H_6_O_2_ compound that was present along with diacetyl (Extended Data Fig. 3) may have also driven ^13^C-enriched emissions, therefore making it difficult to assess genes involved in diacetyl^+^ cycling.

### Metabolomics, substrate composition and metatranscriptomics

To place ^13^C-enriched emissions of acetate, acetone and diacetyl^+^ and the metabolic pathways involved in the cycling of these volatile metabolites into the context of overall microbial responses to drought and substrate availability, we further characterized bulk soil metabolites and microbial gene expression.

Metabolite composition shifted between pre-drought and drought conditions, which separated along the first component (PC1), explaining 44.7 and 62.3% of variation in small (<200–300 Da) compounds measured using ^1^H-NMR (Extended Data Fig. 7a) and relatively larger compounds (>200 Da) characterized using Fourier-transform-ion-cyclotron-resonance MS (FTICR–MS) (Extended Data Fig. 7b), respectively. Small compounds, mainly representing primary metabolites, that decreased during drought included several amino and keto acids (oxiosocaproate, *t*-value = −5.09; alanine, *t*-value = −3.72; formate, *t*-value = −6.25; glycine, *t*-value = −3.12; leucine, *t*-value = −3.16; phenylalanine, *t*-value = −2.71; pyroglutamate, *t*-value = −3.09; pyruvate, *t*-value = −5.31; uracil, *t*-value = −2.94; and valine, *t*-value = −3.34; d.f. = 7, *P* < 0.05, LME), while only trehalose, a common osmolyte produced by bacteria during times of water stress^7^, increased during drought (*t*-value = 3.00; d.f. = 7, *P* = 0.020, LME) (Supplementary Table 1). Drought can induce increased soil concentrations of trehalose^14,15^, yet the impact on amino acids is variable, with some studies finding an increase^13,14,15^ and some a decrease^44^. While we did not detect non-volatile ^13^C-enriched primary metabolites with ^1^H-NMR, decreased concentrations of amino acids during drought could indicate decreased C allocation to biosynthesis of biomass or enhanced degradation of proteins.

For relatively larger compounds, representing microbial metabolism and substrate availability, recalcitrant lignin-, condensed hydrocarbon- and tannin-like compounds increased (*t*-values = 4.17, 4.16, 2.43, respectively; d.f. = 35, *P* < 0.05, LME) and bioavailable protein- and amino-sugar-like compounds, along with lipid-like compounds, decreased during drought (*t*-values = −5.37, −3.92, −5.44, respectively; d.f. = 35, *P* < 0.05, LME) (Extended Data Fig. 7b,c). One possible explanation for this pattern is that microbes preferentially consumed bioavailable compounds during drought, while there was a concurrent decrease in labile C replenishment from root exudates from drought-stressed plants^29^.

Next, we looked at overall shifts in microbial metabolic pathways of co-expressed gene modules using weighted gene co-expression network analysis (WGCNA). We identified a total of nine modules, four of which were associated with condition (pre-drought or drought) and acetate-C2 and acetone-C2 effluxes (Extended Data Fig. 8a,b).

**Fig. 4:**
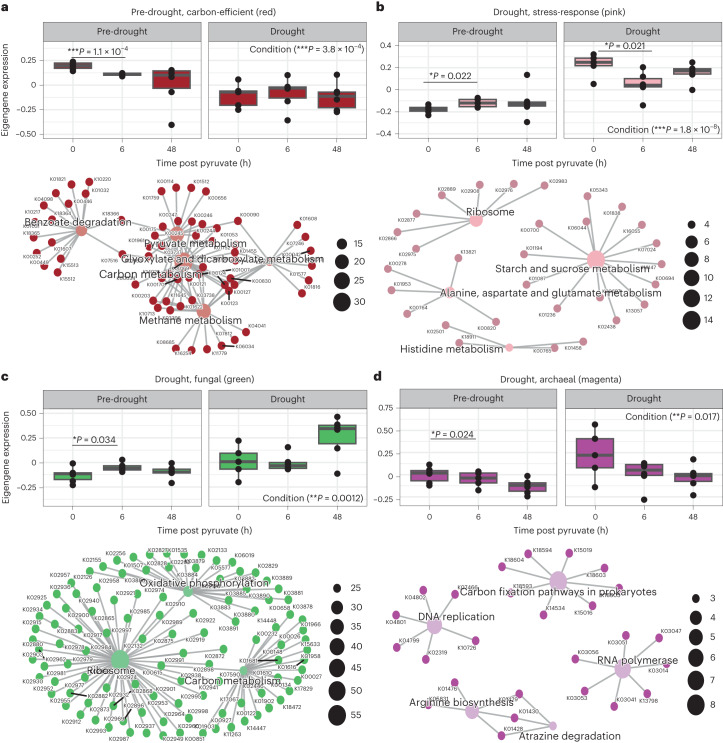
Expression of pre-drought and drought-associated gene modules and their networks of enriched KEGG metabolic pathways. **a**–**d**, Top: eigengene expression at 0, 6 and 48 h post pyruvate addition during pre-drought (0 and 48 h (*n* = 6), 6 h (*n* = 5)) and drought (6 and 48 h (*n* = 6), 0 h (*n* = 5)) conditions for the subset of modules: carbon-efficient, red (**a**); stress-response, pink (**b**); fungal, green (**c**) and archaeal, magenta (**d**). Expression values are arbitrary units. Each point represents one sample; boxes represent Q1–Q3 with centre line indicating median, and bars extend to maximum and minimum values, excluding outliers. Bottom: metabolic network of enriched KEGG pathways within the indicated module. Central nodes represent the pathway and each branch represents the KO group within that pathway. *P* values in **a**–**d** are from linear mixed effects models between pre-drought and drought (as indicated after ‘Condition’) or 6 or 48 h vs 0 h (as indicated by lines between timepoints); *P < 0.05, **P < 0.01, ***P < 0.001. Source data

The pre-drought-associated red module was enriched in C-cycling pathways. The association of the red module with pre-drought conditions was demonstrated by its negative correlation with (*r* = −0.6, *P* = 5.0 × 10^−4^, Pearson correlation coefficient (PCC)) (Extended Data Fig. 8b) and downregulation during (*t*-value = −4.24, d.f. = 30, *P* = 3.8 × 10^−4^, LME) (Fig. 4a) drought. The enriched central C metabolic pathways interwoven within the red module included C metabolism, butanoate metabolism (includes acetone and diacetyl cycling), and pyruvate and methane metabolism (both include acetate cycling) (Fig. 4a). These overlapping central C metabolic pathways suggest efficient use of intermediate C compounds produced, including volatile intermediates such as acetone and acetate, and are supported by negative correlations with acetate-C2 and acetone-C2 efflux (*r* = −0.32 (*P* = 0.090, not significant (NS)) and −0.47 (*P* = 0.010), respectively; PCC) (Extended Data Fig. 8b). Therefore, efficient C cycling in the red module led to rapid consumption of metabolic intermediates, preventing losses to the atmosphere under moist conditions.

The drought-associated pink module played a role in osmotic-stress adaptation, quinone production and acetate/acetone biosynthesis. The pink module was positively correlated with (*r* = 0.8, *P* = 1.0 × 10^−7^, PCC) (Extended Data Fig. 8b) and upregulated during (*t*-value = 7.59, d.f. = 30, *P* < 1.8 × 10^−8^, LME) (Fig. 4b) drought, and it was also potentially involved in acetate and acetone production, as indicated by its positive correlations with acetate-C2 and acetone-C2 efflux (*r* = 0.29 (*P* = 0.010, NS) and *r* = 0.43 (*P* = 0.020), PCC) (Extended Data Fig. 8b) and inclusion of the acetate-producing *poxB* and *poxL* genes. Furthermore, the pink module was enriched in starch and sucrose metabolism; alanine, aspartate and glutamine metabolism; and ribosome pathways (Fig. 4b). Biosynthesis of trehalose within the starch and sucrose metabolism pathway corresponds to the increase in trehalose concentrations observed during drought (Extended Data Table 1). At the sub-pathway level, biosynthesis of ubiquinone, a type of quinone, was also enriched, indicating a possible association with osmotic stress. We hypothesize two explanations that connect quinone biosynthesis, *poxB* expression and acetate biosynthesis as stress-response mechanisms during drought. First, a link between the electron transport chain and osmotic regulation is possible. As an immediate microbial response to osmotic stress, microbes actively pump in K^+^, which then promotes trehalose biosynthesis^45^. A supercomplex of H^+^-ATPase and K^+^ pumps may form in the cellular membrane as part of the electron transport chain^46^. Therefore, increased production of quinone and transport of electrons to the electron transport chain could be linked with K^+^ influx, leading to further osmotic adjustment. Second, due to the high demand for protective biomolecule synthesis (that is, trehalose), there is a high demand for energy production during drought, which may be limited by lower TCA cycle activity (Fig. 2j). To address this need, *poxB* may act as a bacterial PDH bypass route for production of acetyl-CoA (in combination with *acs*)^47^, the precursor to many downstream secondary metabolites^48^ (Fig. 3a). While experiments in *Corynebacterium* found that the PDH bypass route was not essential for growth^38^, *poxB* contributes to the aerobic growth efficiency in glucose-limited conditions in *E. coli*^47^. It is, therefore, possible that under times of stress or within different bacterial species, this route improves fitness by increasing acetyl-CoA production to meet energy demands.

The drought-associated green and magenta modules were enriched in fungal and archaeal metabolic pathways, respectively. Both modules were significantly correlated with (*r* = 0.54 and 0.36, respectively; *P* ≤ 0.05, PCC) (Extended Data Fig. 8b) and upregulated during (*t*-value = 3.94 and 2.26, respectively; d.f. = 30, *P* < 0.05, LME) (Fig. 4c,d) drought; however, while the green module was positively correlated with acetate-C2 and acetone-C2 (*r* = 0.62 and 0.77, respectively; *P* < 0.001, PCC) (Extended Data Fig. 8b) and contained the acetate-cycling genes *ACH1* and *adc* indicating its association with acetate and acetone efflux, the magenta module showed no correlation with ^13^C-enriched VOC emissions or genes of interest. The abundance of fungal genes within the green module’s enriched KEGG pathways indicates a potential role for fungi in the drought response; specifically, the green module was enriched in genes encoding for eukaryotic ribosomal subunits within the ribosome pathway, as well as eukaryotic-specific enzymes (NADH:ubiquinone oxidoreductase and F-type ATPase) within the oxidative phosphorylation pathway (Fig. 4c) (KEGG pathway database^49^). Therefore, the association between the green module and acetate and acetone efflux, as well as fungal-specific genes, suggests a potential role of fungi in acetate and acetone fermentation during drought. While previous studies have found that fungal communities are generally more resistant to stresses, such as drought, compared with bacteria^50–52^, here we did not see a significant increase in total active fungi during drought based on taxonomic classification (0.16 ± 0.7 and 1.1 ± 0.3% in relative abundance during pre-drought and drought conditions, respectively; *t*-value = 0.63, d.f. = 136, *P* = 0.52, LME); however, specific fungal taxa shifted in activity during drought, with *Ascomycota* increasing on average from 44.3 to 68.6% of the fungal community during drought (*t*-value = 3.02, d.f. = 31, *P* = 0.0050, LME) (Extended Data Fig. 4e), with *Fusarium* comprising a majority of this phylum particularly during drought at 48 h post pyruvate addition (52%). Specific studies examining fungal production of acetone are very limited; however, acetone production by *Penicillium brevicompactum* has been detected^53^, and *Fusarium* spp. have been found to produce several volatiles, including acetone and acetate^54^. In contrast, the magenta module was enriched in pathways specific to archaea. For example, the enriched DNA replication and RNA polymerase pathways (Fig. 4d) included polymerases specific to archaea. Despite the association between the drought-associated magenta module and drought, there was no concurrent increase in active archaeal abundance during drought (*t*-value = 0.87, d.f. = 276, *P* = 0.37, LME) (Extended Data Fig. 4d). Collectively, these results suggest that microbial acetate and acetone biosynthesis during drought may be associated more with fungal rather than archaeal metabolic activity, and we suggest further studies to experimentally test this hypothesis.

## Discussion

Here we show how microbially mediated soil C allocation strategies shift from typical (pre-drought) to drought conditions using a combination of ^13^C-pyruvate labelling and multi-omics in a unique controlled-drought experiment performed within an enclosed tropical rainforest. We found a distinct subset of emitted volatiles affected by drought that were formed from pyruvate, hence our findings are representative of pyruvate-dependent volatile emissions from forest floor areas that are not covered by understory vegetation. Under typical pre-drought conditions, there was a balance of C allocated to biosynthesis vs energy, and we were able to track ^13^C into several volatile compounds, all representing primary metabolic intermediates (acetate, acetone and diacetyl^+^). We hypothesize that most of the C was allocated to the biosynthesis of biomass as well, because the ^13^C-enriched volatile intermediates only made up a tiny fraction of the total C allocated to biosynthesis. Therefore, production of these volatiles was matched with immediate recapturing of these molecules during efficient C cycling and utilization. During the stressed conditions imposed by drought, there was a disruption to C allocation pathways. While the balance between C allocated to biosynthesis vs energy remained constant compared to pre-drought conditions, biosynthesis shifted (from presumably biomass) to stress biomolecules, including trehalose and quinone. Furthermore, metabolic pathway shifts led to higher release of ‘leaky’ central metabolites (indicated by increased emissions of ^13^C-enriched acetate, acetone and diacetyl^+^), enhanced by C allocation to VOC production (that is, acetate) and reduced capacity to consume and recoup VOC-C (that is, acetone)^55^. Notably, this decreased C cycling efficiency during drought could be amplified by increased air-filled pore space, isolating microbial communities to microhabitats within the soil matrix that are cut off from an aqueous flow of non-volatile metabolites and substrates^12^. This signifies that during drought, microbes switch from investing in more-stable carbon pools such as biomass^9^ to stress compounds that are prone to be quickly utilized by microbes upon rain rewet^56^. Overall, microbial survival responses to drought shift soil C cycling by interrupting C storage pathways, increasing C allocation to VOCs and loss in efficiency of recouping C from volatile intermediates, thus indicating potential shifts in soil carbon fate.

## Methods

### Biosphere 2 tropical rainforest drought experiment

Biosphere 2 in Oracle, Arizona is a 12,700 m^2^ steel and glass-enclosed building harbouring five distinct biomes, including a 1,900 m^2^ tropical rainforest—an enclosed ecosystem with variable topography and microhabitats with the ability to control rain and temperature inside, providing an optimal setting for studying drought effects^57–59^. This artificial tropical rainforest harbours approximately 70 species of trees and shrubs forming a canopy and understory, with soils that represent biogeochemical cycles present in natural rainforests^60^. In late 2019, a 65-day drought experiment was conducted as part of the Water, Atmosphere and Life Dynamics (WALD) campaign^29^. During ambient (pre-drought) conditions, rainfall events were simulated by spraying 15,000 l of irrigation water from the top of the Biosphere 2 tropical rainforest at a frequency of 3 d a week, with the last rainfall event before the drought occurring on 7 October 2019. During the drought, ambient temperatures were maintained between 20 and 26.7 °C in the lowland region. For more detailed characterization of the WALD drought experiment, please see ref. ^29^.

### Position-specific (C1 and C2) ^13^C-pyruvate labelling

Bulk **s**oil was labelled with position-specific (C1 or C2) ^13^C-pyruvate (henceforth referred to as ^13^C1-pyruvate and ^13^C2-pyruvate, respectively) to track C allocation into CO_2_ and VOCs—a method adapted from one used in plants^61^. Similar ^13^C-pyruvate labelling was performed in other regions of the tropical rainforest during the WALD campaign, including plant roots^62^ and leaves^63^. Three replicates each of ^13^C1-pyruvate or ^13^C2-pyruvate labelling were performed per site (Site 1, Site 2 and Site 3; *n* = 6 per site), representing a vast expanse of the lowland region (Extended Data Fig. 1a) within soil chambers (Extended Data Fig. 1b) before (12–19 September) and during (7–19 November) drought. The night before labelling, two automatic chambers were placed onto the corresponding soil collars, which contained no plants but might have had small amounts of fine roots and were covered with a rain-out shelter. Each morning, these collars were labelled at around 10:00 by placing a 5 × 5 cm stencil with 1 × 1 cm openings into one side of each chamber (placed on different sides of the chamber during pre-drought and drought) and adding 100 μl of ^13^C1-pyruvate or ^13^C2-pyruvate solution (40 mg ml^−1^) (Cambridge Isotopes, CLM-8077-PK and CLM-8849-PK) to each opening to a depth of 1 cm (25 injections), for a total of 100 mg (Extended Data Fig. 1b). The stencil was then removed before soil chamber gas measurements.

Soil moisture and temperature were measured using a portable probe and LabQuest viewer (Vernier). For the pre-drought condition, soil moisture and temperature data were collected on 1 and 9 October and for drought on 11 and 18 November for a subset of collars (P11, P21, P23, P32 and P33 (Site # (P1, P2 or P3), replicate # (1, 2 or 3)), except for 18 November when all collars from the experiment were tested (see Extended Data Fig. 1a). Soil moisture measured near the labelling sites decreased from 26.0 ± 6.9 to 13.8 ± 2.6% (*P* < 0.001) between pre-drought and drought conditions, respectively. Soil temperature did not change significantly and ranged from 23.0 ± 0.6 during pre-drought to 23.3 ± 1.2 °C during drought.

### Continuous monitoring of VOCs and CO_2_

Before labelling, collars were measured at typical temporal resolution (~2 h) overnight. To capture any rapid changes in gas fluxes due to changes in microbial activity after pyruvate addition, measurement intervals were increased to high frequency (30 min) directly before labelling. After gas fluxes were expected to equilibrate, approximately 8 h post pyruvate labelling (~18:00), measurement intervals were decreased to low frequency (50 min) and remained at this frequency until measurements were stopped at 48 h post labelling, resulting in ~64 measurements for each soil collar. For each measurement, the automatic chamber closed over the collar for a total of 10 min (pre-purge, 2.5 min; measurements, 6.5 min; post-purge, 1 min). Fluxes were measured using an automated multiplexed Licor soil flux system (Licor 8100, Li-8150 16-port multiplexer and Lic 8100-104 long-term chambers with opaque lids, Licor). The system was coupled to a Picarro G2201-i analyser (Picarro) to measure CO_2_ and isotopic composition and a proton-transfer-reaction time-of-flight mass spectrometer (PTR–TOF 8000, Ionicon) for VOCs (including ^13^C-VOCs). The PTR–ToF–MS sampled the sub-flow from the soil flux system at 30 standard cubic centimeters per minute (sccm). Perfluoroalkoxy tubing was used for the soil flux system, for the subsampling line and for the PTR inlet, with the aim to minimize the release and the retention of the VOCs from and on the tubing surface^64^. The PTR settings were as follows: inlet temperature was 60 °C, drift voltage was 600 V, drift temperature was 60 °C and drift pressure was 2.2 mbar, resulting in an *E*/*N* (*E* being the electric field strength and *N* the sample gas number density) ratio of 137 Td (Townsend). The time resolution was 10 ms and the mass range was up to 500 amu. The PTR–ToF was operated in the H_3_O^+^ mode, therefore only compounds having proton affinity higher than water (697 kJ mol^−1^) underwent proton-transfer reactions and could be detected on their protonated mass to charge ratio (*m*/*z*), which includes most VOCs. Measured ions were attributed to chemical formulae and specific chemical species based on the exact protonated *m*/*z*. PTR–TOF data were processed using the software PTRwid^65^. To account for possible variations in reagent ion signals, measured ion intensities were normalized to the H_3_O^+^ counts in combination with the water-cluster ion counts^66^. At midnight, automatic calibrations were performed using standard gas cylinders containing different multi-VOC component calibration mixtures in Ultra-High Purity (UHP) nitrogen (Apel-Riemer Environmental). For a detailed description of the calibration setup, see ref. ^29^. The concentrations of compounds included in the standard were calculated with an uncertainty of ≤23%. Concentrations of compounds not included in the calibration standard cylinders were calculated by applying the kinetic theory of proton-transfer reaction^67,68^ with an uncertainty of ≤50%.

Select additional soil experiments were conducted with a Vocus PTR–ToF (TOFWERK)^69^ coupled to a custom-built gas chromatograph (GC) (Aerodyne Research)^70^. The GC contained an integrated two-stage adsorbent-based thermal desorption preconcentration system for in situ collection of VOCs before separation on the chromatographic column. For preconcentration, a multibed (Tenax TA/Graphitized Carbon/Carboxen 1000, Markes International) sorbent tube was used for the first stage of sample collection; this tube was then subjected to a post-collection water purge before the sample was thermally desorbed to a multibed focusing trap (Tenax, Carbopack X, Carboxen 1003, Markes International) before injection onto the GC column. For this study, the GC was equipped with a 30-m Rxi-624 column (Restek, 0.25 mm i.d., 1.4 µm film thickness) which resolves non- to mid-polarity VOCs in the C_5_–C_12_ volatility range before ionization in the PTR detector. The GC–PTR sampled from in situ soil gas probes^71^ on an alternating timed schedule. The GC–PTR can speciate structural isomers and help identify some unknowns by matching to calibrated retention times.

### CO_2_ data analysis

CO_2_ fluxes and their isotopic composition were calculated on the basis of data from the Picarro isotope analyser. Fluxes were calculated with linear and exponential models fitted to each individual chamber measurement. A deadband of 30 s was used for each measurement to allow for mixing in the chamber. The linear models were calculated on the basis of the first 120 s and for the exponential model, the full closure period of 6.5 min was used. Fluxes were quality controlled visually. Fluxes based on the exponential fit were used preferentially but were replaced by the linear-fit flux where necessary. The isotopic composition was calculated on the basis of the individual efflux rates of the ^12^C-CO_2_ and ^13^C-CO_2_ isotopologues. These are reported separately by the gas analyser and linear fits based on the first 120 s were used to calculate efflux rates. Due to the high enrichment of the labelled soil CO_2_ efflux, this method was found to be more reliable compared with conventional Keeling-plot approaches. The isotopic composition of the CO_2_ efflux was then calculated from the ratios of ^12^C-CO_2_ to ^13^C-CO_2_ efflux rates and normalized to the Vienna Pee Dee Belemnite (VPDB) scale (*δ*^13^C_CO2_ = ((^13^C/^12^C)_CO2_)/((^13^C/^12^C)_VPDB_) – 1). C isotope fluxes were quality controlled visually for each individual chamber and outliers removed manually.

^13^C-CO_2_ emitted from chambers that received ^13^C1-pyruvate is formed as C is decarboxylated via pyruvate dehydrogenase (PDH) to form acetyl-CoA or via an alternate decarboxylation reaction leading to biosynthesis of compounds (for example, biomass, secondary metabolites, VOCs), while ^13^C-CO_2_ emitted from chambers that received ^13^C2-pyruvate is primarily formed during decarboxylation in the TCA cycle during energy production (Fig. 1) (modified from refs. ^26,27^). Using this concept, we can approximate both total and relative (proportion of total) C allocated to biosynthesis by calculating ^13^C-CO_2-C1_ − ^13^C-CO_2-C2_ and ^13^C-CO_2-C1_/(^13^C-CO_2-C1_ + ^13^C-CO_2-C2_), respectively, where ^13^C-CO_2-C1_ and ^13^C2-CO_2-C2_ are efflux from chambers that received ^13^C1-pyruvate or ^13^C1-pyruvate, respectively. Within each site, there were three sets of ‘C1’ and ‘C2’ chambers located next to each other (Extended Data Fig. 1), and the calculations above were made for each set. To facilitate calculation of total and relative C allocation over time, continuous flux data were binned and averaged across 0–3 (3 h), 3–6 (6 h), 6–12 (12 h), 12–18 (18 h), 18–24 (24 h), 24–30 (30 h), 30–36 (36 h), 36–42 (42 h) and 42–48 (48 h). These calculations are approximations due to two reasons. First, heterotrophic and autotrophic CO_2_ fixation, including anaplerotic reactions^31,72^, leads to ^13^C-CO_2_ being immediately re-consumed after production, causing an underestimate of actual ^13^C-CO_2_ emissions by up to 5.6% (based on soil CO_2_ fixation rates calculated from a forest soil^31^). Second, due to anaplerotic CO_2_ assimilation, some ^13^C from ^13^C1-pyruvate may have entered the TCA cycle and been released as ^13^C-CO_2_, causing an overestimate of biosynthesis. However, assuming constant CO_2_ fixation routes in pre-drought and drought conditions, this would not have impacted the conclusions we made from our calculations.

### VOC data analysis

The isotopic composition of VOC flux rates was calculated by applying the linear model to calculate the rate of change in the fractional abundance of ^13^C (^13^C-VOC/(^13^C-VOC + ^12^C-VOC)). For each 6.5 min chamber measurement, a deadband of 30 s was used to allow for mixing in the chamber and the linear model was applied to the successive 10 data points collected at 10 s intervals. To identify pyruvate metabolism pathways, we focused on those VOCs that showed ^13^C isotopic enrichment in their emissions. To help clarify metabolic pathways and direction of reactions, we also considered the fluxes of VOCs independent of their ^13^C-enrichment and metabolite concentrations immediately up- or downstream of the ^13^C-enriched VOCs (Fig. 3a). For a complete analysis of all VOCs that showed changes in flux patterns across the drought experiment, see ref. ^73^.

### Cumulative effluxes and distribution of ^13^C from pyruvate

Cumulative ^13^C-CO_2_ and ^13^C-VOC effluxes from 0 to 48 h post ^13^C-pyruvate injection were calculated using the area under the curve ‘auc’ function within the FLUX package (v.0.3)^74,75^ in R on the basis of the total amount of ^13^C-pyruvate added, we could determine what percentage of total ^13^C ended up in CO_2_ or VOCs.

### Soil sample collection and processing

For metagenomics, metatranscriptomics and metabolomics, soil samples were collected just before ^13^C-pyruvate labelling (0 h) then at 6 and 48 h post labelling. For 0 h collection, soil (~ 8 g) was collected directly outside of the stencil using a 2.25 cm diameter sterile metal ring pushed into the soil to 2 cm depth and placed into a sterile 50 ml tube. For 6 and 48 h collections, soil samples were collected using the same method as for 0 h, but inside the metal frame where pyruvate labelling occurred. After each soil collection, samples were immediately brought to the lab and allocated for different downstream analyses: 1 g stored at −20 °C for metabolomics and 2 g in Lifeguard soil preservation solution (Qiagen, 12868–1000) stored at −80 °C for DNA/RNA extractions.

RNA and DNA were co-extracted from 1 g of soil using the RNeasy Powersoil Total RNA kit (Qiagen, 12866–25) coupled with the RNeasy Powersoil DNA Elution kit (Qiagen, 12867–25) following the manufacturer’s protocol and eluted in 100 μl of kit-provided elution buffer. RNA and DNA concentrations and quality were measured using a Qubit 4 fluorometer (Thermo Fisher) and NanoDrop spectrophotometer (Thermo Fisher). RNA was further treated with DNAse (DNAse Max, Qiagen, 15200–50) to remove any DNA contamination. Total RNA and DNA were sent to the Joint Genome Institute (JGI; Berkeley, California) for library prep and sequencing.

Water extractions were performed on samples for NMR, followed by solid phase extraction (SPE) for FTICR–MS. Water extraction procedures for bulk metabolite characterization were followed^76–78^. Briefly, 1 g of soil and 5 ml of double deionized water were vortexed in a 15 ml centrifuge tube, sonicated for 2 h at 21 °C, then centrifuged for 20 min. One ml of water-extractable organic C (WEOC) was removed and stored at −80 °C for NMR analysis at the Pacific Northwest National Laboratory (PNNL), while 4 ml of WEOC was saved for downstream SPE.

SPE is an extraction technique used to clean and concentrate samples for the isolation and analysis of organic compounds^79^. Four ml of WEOC were acidified to pH 2 with 1 M HCl, then passed through methanol (MeOH)-preactivated Bond Elut PPL (Priority PolLutant, Agilent, 12255002) barrel cartridges that contain a macroporous styrene-divinylbenzene crosslinked polymer to retain polar organic compounds, with a vacuum set no higher than −5 psi. Next, the cartridges were washed three times with 9 ml 0.01 M HCl to remove impurities, air dried with a pressure air hose for 2–3 min and finally rinsed with 1.5 ml MeOH in a slow dropwise flow rate into 2 ml vials. The eluate was stored at −80 °C and sent to PNNL for FTICR–MS analysis.

### Metabolomics (NMR and FTICR) measurement and analysis

^1^H-NMR bulk metabolite characterization was performed on WEOC. Samples (180 µl) were combined with 2,2-dimethyl-2-silapentane-5-sulfonate-d_6_ (DSS-d_6_) in D_2_O (20 µl, 5 mM) and thoroughly mixed before transfer to 3 mm NMR tubes. Resonances corresponding to ^13^C labelling were identified by visual inspection, comparing labelled spectra to unlabelled spectra. Once differences were identified, the molecular location and quantification of ^13^C incorporation were determined by J-coupling pattern analysis and the ‘linefitting’ tool in MestReNova (v.14.2.014)^80^, respectively; however, ^13^C-labelled metabolites could not be identified in our samples. NMR spectra were acquired on a Bruker Advance III spectrometer operating at a field strength of 17.6 T (^1^H ν_0_ of 750.24 MHz) and equipped with a 5 mm Bruker TCI/CP HCN (inverse) cryoprobe with *Z*-gradient and at a regulated temperature of 298 K. The one-dimensional (1D) ^1^H spectra were acquired using a Nuclear Overhauser Effect Spectroscopy (NOESY) pulse sequence (noesypr1d). The 90° H pulse was calibrated before the measurement of each sample with a spectral width of 12 ppm and 1,024 transients. The NOESY mixing time was 100 ms and the acquisition time was 4 s, followed by a relaxation delay of 1.5 s during which presaturation of the water signal was applied. Time domain free induction decays (72,114 total points) were zero filled to 131,072 total points before Fourier transform, followed by exponential multiplication (0.3 Hz line-broadening) and semi-automatic multipoint smooth segments baseline correction. Chemical shifts were referenced to the ^11^H methyl or ^13^C signal in DSS-d_6_ at 0 ppm. The 1D ^11^H-NMR spectra of all samples were processed, assigned and analysed using Chenomx NMR suite 9.2 (Chenomx) with quantification of spectral intensities of compounds in the Chenomx library relative to the internal standard. Candidate metabolites present in each of the complex mixtures were determined by matching chemical shift, J-coupling and intensity information of the experimental signals against signals of the standard metabolites in the Chenomx library. Signal to noise ratios (S/N) were measured using MestReNova with the limits of quantification and detection equal to an S/N of 10 and 3, respectively. Standard 2D experiments such as ^11^H/^13^C - heteronuclear correlation (HSQC) or 2D ^11^H/^11^H Total Correlation spectroscopy (TOCSY) further aided corroboration of several metabolite identifications where there was sufficient S/N.

A 12-Tesla Bruker FTICR mass spectrometer was used to collect high-resolution mass spectra of SPE-filtered WEOC by direct injection for secondary metabolite characterization. Approximately 100 µl of water extract was mixed with MeOH (1:2) before injection to enhance ionization. Samples were directly injected into a standard Bruker electrospray ionization (ESI) source. The instrument settings were optimized by tuning on a Suwannee River fulvic acid standard (International Humic Substances Society), and the instrument was flushed between samples using a mixture of water and MeOH. Blanks (HPLC grade MeOH) were analysed at the beginning and end of the day to monitor potential carry over from one sample to another. The ion accumulation time varied to account for differences in C concentration between samples. For each sample, 144 individual scans were averaged and internally calibrated using an organic matter homologous series separated by 14 Da (CH_2_ groups). The mass measurement accuracy was <1 ppm for singly charged ions across a broad *m*/*z* range (100–1,000 *m*/*z*). The mass resolution was ∼240 K at 341 *m*/*z*. The transient was 0.8 s. Data Analysis software (BrukerDaltonik v.4.2) was used to convert raw spectra to a list of *m*/*z* values, applying the FTICR–MS peak picker module with an S/N threshold set to 7 and absolute intensity threshold set to the default value of 100. Putative chemical formulae were then assigned using Formularity software^81^ on the basis of the following criteria: S/N > 7, mass measurement error <1 ppm and taking into consideration the presence of C, H, O, N, S and P and excluding other elements^82^. To ensure consistent formula assignment and eliminate mass shifts that could impact formula assignment, all sample peak lists were aligned to each other. The following rules were implemented to further ensure consistent formula assignment: (1) picking formulae with the lowest error between predicted and observed *m*/*z* and the lowest number of heteroatoms and (2) requiring the presence of at least four oxygen atoms for the assignment of one phosphorus atom^82^. The chemical character of thousands of peaks in each sample’s ESI FTICR–MS spectrum was evaluated using van Krevelen diagrams, with biochemical compound classes reported as relative abundance values on the basis of counts of C, H and O as follows: lipids (0 < O:C ≤ 0.3 and 1.5 ≤ H:C ≤ 2.5), unsaturated hydrocarbons (0 ≤ O:C ≤ 0.125 and 0.8 ≤ H:C < 2.5), proteins (0.3 < O:C ≤ 0.55 and 1.5 ≤ H:C ≤ 2.3), amino sugars (0.55 < O:C ≤ 0.7 and 1.5 ≤ H:C ≤ 2.2), lignin (0.125 < O:C ≤ 0.65 and 0.8 ≤ H:C < 1.5), tannins (0.65 < O:C ≤ 1.1 and 0.8 ≤ H:C < 1.5) and condensed hydrocarbons (aromatics; 0 ≤ 200 O:C ≤ 0.95 and 0.2 ≤ H:C < 0.8)^78^. Analysis of FTICR data was performed using MetaboDirect (v.0.2.7)^83^ to create profiles of biochemical compound classes and principal component analysis (PCA) plots.

### Metagenomics and metatranscriptomics sequencing and analysis

Metagenome and metatranscriptome libraries were created at the JGI. Plate-based DNA library preparation for Illumina sequencing was performed on the PerkinElmer Sciclone NGS robotic liquid handling system using the Kapa-HyperPrep library preparation kit (Kapa Biosystems). Sample genomic DNA (200 ng) was sheared to 300–500 bp using a Covaris LE220 focused-ultrasonicator. The sheared DNA fragments were size selected by double-SPRI, then the selected fragments were end-repaired, A-tailed and ligated with Illumina-compatible sequencing adaptors (from IDT) containing a unique molecular index barcode for each sample library.

For metatranscriptome libraries, ribosomal (r)RNA was removed from 100 ng of total RNA using Qiagen FastSelect 5S/16S/23S for bacterial rRNA depletion (and additional FastSelect plant and/or yeast rRNA depletion) (Qiagen) with RNA blocking oligo technology. The fragmented and rRNA-depleted RNA was reverse transcribed to create first-strand complementary (c)DNA using Illumina TruSeq Stranded mRNA Library prep kit (Illumina), followed by the second-strand cDNA synthesis which incorporated dUTP to quench the second strand during amplification. The double-stranded cDNA fragments were then A-tailed and ligated to JGI dual indexed Y-adapters, followed by an enrichment of the library by 10 cycles of PCR. Metatranscriptomics of sample P35SSC1_191108_c were not completed due to librarly prep and sequencing issues.

The prepared libraries were quantified using KAPA Biosystems’ next-generation sequencing library qPCR kit and run on a Roche LightCycler 480 real-time PCR instrument. Sequencing of the flowcell was performed on the Illumina NovaSeq sequencer using NovaSeq XP V1.5 reagent kits and S4 flowcell, following a 2 × 151 indexed run recipe.

Sequence filtering, assembly, mapping and annotation were performed at JGI^84^. BBDuk (v.38.94), included in the BBtools package^85^, was used to trim reads that contained adapter sequences, homopolymers of G’s of the size 5 or more at the ends of reads and reads where quality dropped to 0. BBDuk was also used to remove reads that contained 1 or more ‘N’ bases, had an average quality score across the read of <10, or had a minimum length of ≤51 bp or 33% of the full read length. Reads were mapped with BBMap (v.38.44), included in the BBtools package, to masked human, cat, dog and mouse references at 93% identity, and common microbial contaminants were removed for downstream analysis. Filtered reads were error corrected before assembly using bbcms (v.38.90), included in the BBtools package and assembled using metaSPAdes (v.3.15.2)^86^ with a minimum contig of 200 bp (for metatranscriptome libraries, there was an additional removal of rRNA during filtering and assembly was performed using MegaHit (v.1.2.9)^87^). Filtered reads were then mapped back to contigs using BBMap to obtain coverage information. See Extended Data Table 2 for sequencing depth and mapping statistics. Feature prediction was next performed on assembled contigs by using tRNAscan-SE (v.2.0.6)^88^ to predict transfer RNAs, INFERNAL (v.1.1.3)^89^ to identify non-coding RNAs and rRNAs, CRT-CLI^90^ to identify CRISPR regions, and Prodigal (v.2.6.3)^91^ and GeneMarkS-2 (v.1.07)^92^ to identify protein-coding genes (CDSs). Functional annotation was performed on CDSs with KEGG Orthology (KO) terms^93^. Taxonomy for each CDS was determined using the best LAST^94^ hits of CDSs, then contigs were classified on the basis of the majority rule (the lowest taxonomic rank that at least 50% of CDSs on the contig matched to^84^). Gene copies per KO were calculated as the average coverage of the contigs each gene was predicted from, multiplied by the number of genes in the KO^84^.

Active metabolic pathways were determined by mapping KOs to KEGG pathways using the KEGG pathway mapper tool^93^. VOC cycling genes were chosen on the basis of their being immediately up- or downstream from the VOC in the metabolic pathway, as well as being downstream from pyruvate. Gene symbols used in the paper are based on the KEGG database. Whether the gene was involved in production or consumption of VOC was assessed by examining the reaction on the KEGG database and on the basis of the literature.

### Statistical analysis

All statistical analyses were performed in R (v.4.0.2)^95^. First, to compare ^13^C-CO_2_ and ^13^C-VOC (acetate, acetone and diacetyl^+^) cumulative fluxes across pre-drought and drought conditions, LME modelling was performed using the LME function in the NLME package (v.3.1)^96^ with ‘Site’ and ‘ID’ (location within each site) included as random variables to control for these confounding factors. Next, LME was used to compare differences in total and relative C allocation to biosynthesis (^13^C-CO_2-C1_–^13^C-CO_2-C2_ and ^13^C-CO_2-C1_/(^13^C-CO_2-C1_ + ^13^C-CO_2-C2_), respectively) between pre-drought and drought conditions. The following model equation was used for both of the above LME analyses (full scripts are included in GitHub at github.com/linneakh/SoilPyruvate):

$$\begin{array}{c}{\rm{lme}}\left(\right.\rm{response} \sim {\rm{Condition}},\\ \,{\rm{random}}={\rm{list}}\left(\right.{\rm{Site}}=-1,\\ \,\left.{\rm{ID}}=-1\right),\\ \,{\rm{data}}={\rm{data}},\\ \,{\rm{weights}}={\rm{varIndent}}({\rm{form}}=-1|{\rm{Condition}})\end{array}$$

WGCNA was performed on metatranscriptomic data to identify modules of co-expressed genes. First, gene copy data were normalized in DESeq2 (v.1.30.1)^97^ using the variance stabilization transformation (VST) method and samples were clustered to detect outliers, which removed only one sample (P15SSCI_191107_c). Next, the normalized data were analysed using the WGCNA package (v.1.7)^98,99^ in R employing the ‘blockwiseModules’ function with the following settings: soft-thresholding power of 6, minimum module size of 80, minimum KME of 0.35 and a ‘signed’ topology overlap matrix. Pearson correlation coefficients (*r*) were calculated between module eigengenes and the following: (1) condition (values for pre-drought and drought set to −1 and 1, respectively) and (2) ^13^C-enriched VOC fluxes (acetate-C2 and acetone-C2) averaged at 0–2, 5–7 and 46–48 h post pyruvate addition to correspond with metatranscriptome soil collection times of 0, 6 and 48 h, respectively.

To identify expressed genes that were contributing to ^13^C-enriched VOC efflux, PLSR analysis was performed using the pls package (v.2.8)^100^. This multivariate statistical test identifies predictive variables that contribute to a response variable while allowing for collinearity between variables. Three PLSR models were created using the ^13^C-enriched flux (acetate-C2, acetone-C2 and diacetyl^+^-C2) as a single response variable for each model. To match flux measurements to gene expression data, we averaged VOC flux measurements for 0–2, 5–7 and 46–48 h post pyruvate addition to correspond with metatranscriptome soil collection times of 0, 6 and 48 h, respectively. As predictive variables, the VST-normalized gene copy data were used from collars that received ^13^C2-pyruvate (Site2-P4, Site2-P6 and Site3-P4) from across three timepoints post pyruvate injection (0, 6 and 48 h) during pre-drought and drought conditions (*n* = 18). To account for the correlation between acetate-C2 and acetone-C2 at the 0, 6 and 48 h timepoints, which is probably due to the interconnection of the pathways for cycling of these compounds in metabolic pathways, acetate-C2 flux was added as a predictive variable to the acetone model, and acetone-C2 flux was added as a predictive variable to the acetate model. Furthermore, to improve the models, we included soil moisture (%) which likely impacts the movement of VOCs through the soil and emissions to the atmosphere^8^. The optimal number of components for each model was determined by ‘leave one out’ cross-validation, calculating the root mean squared error in cross-validation (RMSECV) using 0–10 components and finding the number of components that had the lowest RMSECV value. The degree of importance of each predictive variable was assessed using the variable importance in prediction (VIP) values, which were calculated from the loadings, weights and scores of each variable^101^ using the plsVarSel package (v.0.9.10)^102^. Additionally, to determine the significance of the model, cross-validated predictive residuals were compared to the residuals of the null model (that is, using the mean of the response variable) using an *F*-test. By comparing *F*-values for each model and their corresponding degrees of freedom, a *P* value could be calculated to determine whether the model was significantly different from the null. To determine which predictive variables significantly contributed to each model, the predictor variable with the lowest VIP was removed and a new model was created. This was repeated until the model with the highest % of variation explained (*R*^2^ × 100) was obtained, indicating the predictive variables that were the largest drivers of the response variables. The variables removed from each model were as follows: *acmB* (K18372) for the acetate model; *acmB* (K18372), *acxC* (K10856), *acxA* (K10855) and *acxB* (K10854) for the acetone model; and *ilvB* (K01652) and *BDH* (K0004) for the diacetyl^+^ model. The optimal numbers of components for each model were determined as follows: 6 components for acetate (*R*^2^ = 0.51, 0.19, 0.16, 0.03, 0.03 and 0.002 (total *R*^2^ = 0.91)), 1 component for acetone (*R*^2^ = 0.76) and 2 components for diacetyl (*R*^2^ = 0.15 and 0.08 (total *R*^2^ = 0.23)), with corresponding RMSECV values of 42.6, 62.5 and 31.6, respectively. This resulted in the following *P* values: 0.50 for acetate, 0.005 for acetone and 0.93 for diacetyl^+^ models.

To find differences in metabolomic composition and metagenomic and metatranscriptomic gene copies between pre-drought and drought conditions, PCA was performed using the built-in R ‘prcomp’ function on log-transformed NMR data for all metabolites, FTICR data collapsed at the compound class level and VST-transformed normalized gene copy data using the prcomp function in base R and plotted with ggplot2 (v.3.4.1)^103^. LME models were created as described above but with only ‘Site’ as a random variable to detect significant differences in all NMR metabolites and FTICR compounds classes between pre-drought and drought conditions. To determine which genes were up- or downregulated during drought, DESeq2 was used to find the log_2_FC and associated *P* values (false discovery rate (FDR) corrected) between pre-drought vs drought conditions while controlling for differences between ‘Sites’.

For WGCNA eigengene expression and taxonomic composition, LME models were created with only ‘Site’ as a random variable to determine significant differences between (1) pre-drought vs drought conditions across all timepoints and (2) 6 and 48 vs 0 h within each condition (pre-drought or drought, using ‘Time’ in place of ‘Condition’ in the model). *P* values reported for Pearson correlations between module eigengenes and ^13^C-enriched VOC fluxes (acetate-C2 and acetone-C2) were corrected for FDR. Enriched KEGG metabolic pathways within each module were calculated using the ClusterProfiler package (v.3.18.1)^104^, with the KEGG reference database set to ‘ko’.

### Reporting summary

Further information on research design is available in the Nature Portfolio Reporting Summary linked to this article.

## Supplementary information

Supplementary Information Supplementary Figs.1–8, and Tables 1 and 2.

Reporting Summary

Supplementary Data 1 Folder containing all raw XML data for FTICR.

Supplementary Data 2 Soil moisture and temperature data.

## Source data

Source Data Fig. 2 dCO_2_ flux, acetate, acetone, diacetyl flux and area under the curve data, and CO_2_ C1-C2 differences.

Source Data Fig. 3 DESeq2 results of VOC cycling genes in Fig. 3.

Source Data Fig. 4 WGCNA eigengene expression for pink, red, green and magenta modules.

Source Data Extended Data Fig. 2 CO_2_ flux and CO_2_ C1-C2 ratios.

Source Data Extended Data Fig 4 metaT/metaG PCA coordinates and metaT/metaG taxonomy.

Source Data Extended Data Fig. 5 DESeq2 results of VOC cycling genes in Extended Data Fig. 5.

**Extended Data Fig. 6:**
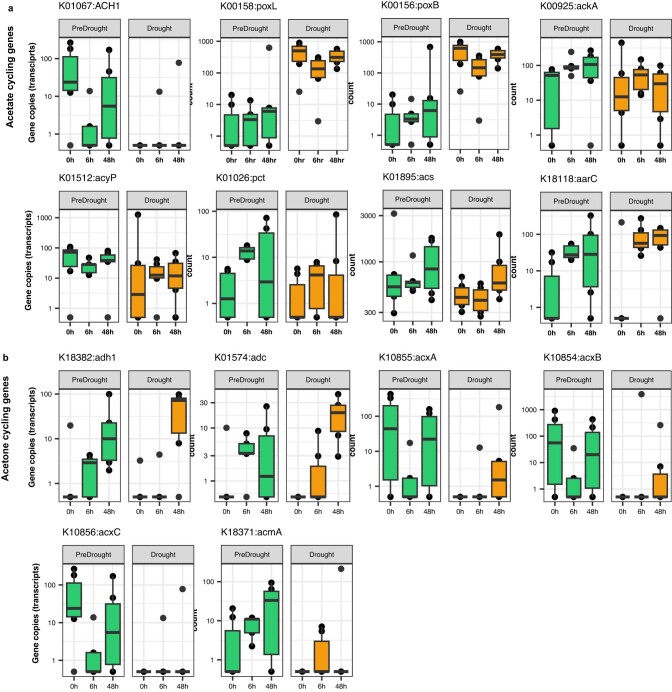
Acetone and acetate cycling gene transcripts. Gene copies (transcripts) of **a)** acetate- and **b)** acetone-cycling genes at 0, 6, and 48 h post pyruvate injection. Bars represent Q1 - Q3 with center line indicating median, and bars extending to maximum and minimum values, excluding outliers, across all chambers and sites (n = 9 each for pre-drought and drought). Source data

Source Data Extended Data Fig. 6, Source Data Extended Data Fig. 8 Gene/transcript count table, sample metadata and trait data for WGCNA.

**Extended Data Fig. 7:**
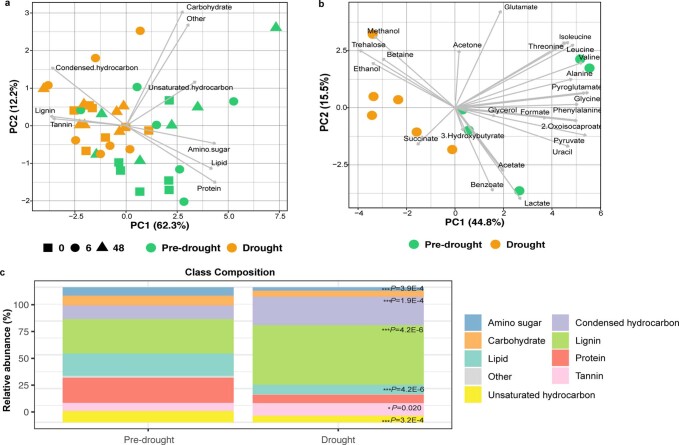
Drought induced a shift in both primary and secondary metabolic composition. **a)** PCA biplot of samples collected during pre-drought (green; 0 h [n = 5]) or drought (orange; 0 h [n = 5] and 6 h [n = 1]) showing relationships between metabolic profiles identified with NMR which were mostly identified as primary metabolites with relatively low molecular weight. Arrows represent the loadings of individual metabolites. **b)** PCA biplot of samples collected during pre-drought (green) or drought (orange) showing relationships between profiles of metabolic classes identified with FTICR-MS, capturing mostly high molecular weight metabolites including secondary metabolites. Arrows represent the loadings of metabolic classes driving these patterns. *P*-values indicate significance of clustering between pre-drought and drought conditions using PERMANOVA on bray-curtis dissimilarity matrix. **c)** Each stacked bar represents the composition of compound classes identified with FTICR-MS for all samples (n = 18 [6 chambers sampled at 0, 6, and 48 h post pyruvate addition] averaged across pre-drought and drought conditions). Exact *P*-values are shown as determined by Linear Mixed Effects Models; *, *P* < 0.05; **, *P* < 0.01; ***, *P* < 0.001. Source data

**Extended Data Fig. 8:**
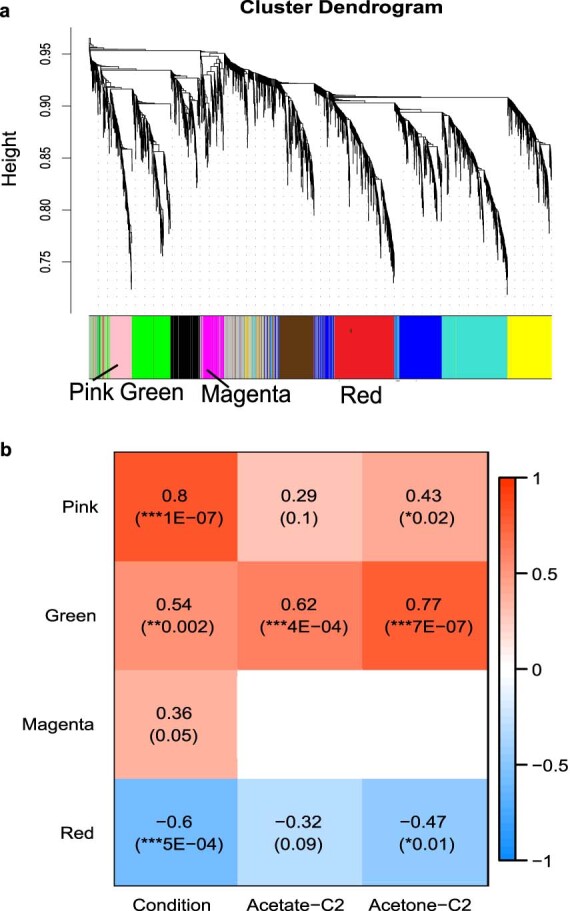
Modules of co-expressed genes that are associated with pre-drought or drought conditions and ^13^C-enrichment of VOCs. **a)** WGCNA cluster dendrogram reveals 9 clusters of co-expressed genes, or modules. The four modules with significant correlations to drought or pre-drought (pink, green, magenta, and brown) are indicated. **b)** Correlations of the pink, green, magenta, and brown module eigengenes with environmental conditions (pre-drought = 0, drought = 1) and efflux of ^13^C-enriched (VOC ^13^C/(^12^C + ^13^C)) acetate and acetone from chambers receiving ^13^C2-pyruvate (acetate-C2 and acetone-C2, respectively) across three time points (0, 6, and 48 h post pyruvate injection; total n = 27 [3 measurements each for each soil collar receiving ^13^C2-pyruvate]). Pearson correlation coefficients are shown with FDR-corrected p-values below in parentheses. White boxes indicate no correlation (*P* < 0.1). *, *P* < 0.05; **, *P* < 0.01; ***, *P* < 0.001. Source data

Source Data Extended Data Fig. 7, Source Data Extended Data Table 1 NMR data and FTICR class compounds.

## Data Availability

The metatranscriptomics and metagenomics sequence data are publicly available through Genbank SRA under the following BioProject IDs: PRJNA980752–PRJNA980834. FTICR, NMR, VOC and CO_2_ data have been deposited at FigShare (10.6084/m9.figshare.20334537). Individual raw.xml files for the FTICR data and soil temperature/moisture data are included as Supplementary Data. Source data are provided with this paper.

## Extended data

**Extended Data Table 1:** Metabolic concentrations (μM) detected using NMR

Compound		Pre-drought					Drought					
Name	Formula	P15-0hr	P24-0hr	P26-0hr	P34-0hr	P35-0hr	P12-Ctrl	P15-0hr	P24-6hr	P26-0hr	P34-0hr	P34-0hr
2-Oxoisocaproate**^P^	C_6_H_10_O_3_	6	5	D	5	D	ND	ND	ND	ND	ND	D
3-Hydroxybutyrate	C_4_H_8_O_3_	ND	ND	ND	ND	D	ND	ND	ND	ND	ND	ND
Acetate	C_2_H_4_O_2_	22	38	30	55	26	19	35	31	31	24	11
Acetone	C_3_H_6_O	ND	D	D	ND	ND	D	ND	ND	ND	ND	D
Alanine**^P^	C_3_H_7_NO_2_	24	21	15	20	17	18	9	13	9	7	9
Benzoate	C_7_H_6_O_2_	ND	ND	ND	D	ND	ND	ND	ND	ND	ND	ND
Betaine	C_5_H_11_NO_2_	ND	ND	ND	ND	ND	D	D	ND	ND	ND	ND
Ethanol	C_2_H_6_O	12	17	21	19	18	28	24	12	22	22	12
Formate***^P^	CH_2_O_2_	17	38	40	23	31	ND	D	6	16	D	11
Glutamate	C_5_H_9_NO_4_	12	12	8	6	10	11	7	10	10	10	6
Glycerol	C_3_H_8_O_3_	6	8	8	6	7	5	6	7	5	8	6
Glycine*^P^	C_2_H_5_NO_2_	12	9	6	8	7	5	D	7	6	D	D
Isoleucine	C_6_H_13_NO_2_	7	7	D	D	D	D	D	D	D	D	D
Lactate	C_3_H_6_O_3_	6	7	D	12	7	D	D	11	D	D	7
Leucine*^P^	C_6_H_13_NO_2_	12	11	7	7	6	7	6	6	6	D	D
Methanol	CH4O	150	375	367	222	326	508	329	203	370	372	322
Phenylalanine*^P^	C_9_H_11_NO_2_	D	D	ND	D	ND	ND	ND	ND	ND	ND	ND
Pyroglutamate*^P^	C_5_H_7_NO_3_	22	19	10	16	9	8	5	12	8	D	D
Pyruvate**^P^	C_3_H_4_O_3_	11	13	10	11	10	D	5	9	5	D	7
Succinate	C_4_H_6_O_4_	ND	ND	ND	ND	ND	ND	D	D	ND	ND	ND
Threonine	C_4_H_9_NO_3_	6	7	D	D	D	D	D	D	D	D	D
Trehalose*^D^	C_12_H_22_O_11_	ND	ND	ND	ND	ND	18	D	7	6	13	D
Uracil*^P^	C_4_H_4_N_2_O_2_	D	5	D	D	D	ND	ND	D	ND	ND	D
Valine*^P^	C_5_H_11_NO_2_	15	13	8	9	8	8	6	8	6	5	5

Samples displayed are a subset of total samples based on the quality of NMR data. Most samples are from 0 h, except for P24 during drought which is from 6 h post pyruvate injection.

ND, not detected (below the level of detection where concentration < 1.9 μM); D, detected (below level of quantification, where concentration 2 - 5 μM)

^P^, Compound higher during pre-drought (Linear Fixed Effect Model (d.f. = 7), where ND and D were given values of 1 and 4 μM respectively, Oxoisocaproate *P*=0.0014, Alanine *P*=0.0074, Formate *P=*4.2E-4, Glycine *P*=0.017, Leucine *P*=0.016, Phenylalanine *P*=0.030, Pyroglutamate *P*=0.018, Pyruvate *P*=0.0011, Uracil *P*=0.022, Valine *P*=0.012)

^D^, Compound higher during drought (Trehalose *P*=0.020)

*, *P*<0.05; **, *P*<0.01; ***, *P*<0.001

**Extended Data Table 2:** Sequence coverage for metagenomics and metatranscriptomics

	Metagenomics			Metatranscriptomics		
Sample	Reads	Bases (Gb)	Mapped to assembly (%)	Reads	Bases (Gb)	Mapped to assembly (%)
P11SSC1_190916_c	142713986	21.4	49.1	124166928	18.0	77.9
P11SSC1_190916_f	224647504	33.6	60.0	134601358	19.5	81.2
P11SSC1_190916_i	155786656	23.3	53.4	101082054	14.5	77.6
P11SSC1_191110_c	227806600	34.1	64.9	82931902	12.0	81.1
P11SSC1_191119_f	132381506	19.8	55.9	107176982	15.4	82.3
P11SSC1_191119_i	257335184	38.5	64.8	76710842	11.0	81.1
P12SS_CTRL_191116_c	135568486	20.3	52.9	92715120	13.2	77.0
P12SS_CTRL_191116_f	103105310	15.4	48.0	146171918	20.8	73.4
P12SS_CTRL_191116_i	89432070	13.4	45.8	85117324	12.1	81.9
P15SSC1_190912_c	141457632	21.2	44.5	95565802	13.6	77.1
P15SSC1_190913_f	136093864	20.4	41.0	102381422	14.5	73.1
P15SSC1_190915_i	116566008	17.5	39.7	105544744	15.0	67.5
P15SSC1_191107_f	126857110	19.0	43.0	22699542	3.2	68.0
P15SSC1_191107_i	141678932	21.2	43.5	104936440	14.9	75.1
P15SSCI_191107_c	131734810	19.7	45.6	106270712	15.0	78.9
P24SSC2_190915_c	131640432	19.7	54.6	93137922	13.3	80.2
P24SSC2_190915_f	108754462	16.3	50.0	145088652	20.6	77.5
P24SSC2_190915_i	143103050	21.4	55.0	120662996	17.1	73.7
P24SSC2_191109_c	122604752	18.4	49.7	124732280	17.6	74.2
P24SSC2_191109_f	145461884	21.8	59.2	79879116	11.5	74.6
P24SSC2_191109_i	101194304	15.2	44.3	72649070	10.2	77.6
P26SSC2_190918_c	138246448	20.7	58.3	76395558	10.8	63.8
P26SSC2_190918_f	110179686	16.5	54.9	94868962	13.4	65.5
P26SSC2_190918_i	112067984	16.8	49.2	116842828	16.7	76.7
P26SSC2_191112_c	98449944	14.7	50.9	85137010	12.0	72.2
P26SSC2_191112_f	119958450	18.0	58.9	78330638	10.9	68.4
P26SSC2_191112_i	175881098	26.3	62.4	97683038	13.7	68.9
P34SSC2_190917_c	132462676	19.8	55.5	71554676	10.2	77.8
P34SSC2_190917_f	101846050	15.3	48.6	183218676	25.8	78.6
P34SSC2_190917_i	127521864	19.1	51.5	85630146	12.0	69.7
P34SSC2_191111_c	154773318	23.2	57.8	83204978	12.0	84.2
P34SSC2_191111_f	134298460	20.1	56.9	78791904	10.8	72.9
P34SSC2_191111_i	112046872	16.8	53.5	68499682	9.9	80.1
P35SSC1_190914_c	112270418	16.8	56.0	16633060	2.3	65.2
P35SSC1_190914_f	112035746	16.8	55.5	na	na	na
P35SSC1_190914_i	128627610	19.3	60.0	14394504	2.0	37.3
P35SSC1_191108_c	195950226	29.3	64.4	13623088	1.9	59.7
P35SSC1_191108_f	128984880	19.3	56.8	97460190	13.5	72.1
P35SSC1_191108_i	109718370	16.4	55.1	86907854	12.1	74.3

a, 0 h; f, 6 h; i, 48 h

na, Metatranscriptomics of sample P35SSC1_191108_c were not completed due to sequencing issues

Read counts are post-filtering (as described in the methods section).

## Code availability

All code used in data analysis and to create figures is available at www.github.com/linneahkh/SoilPyruvate.
