# Author Correction: Drought re-routes soil microbial carbon metabolism towards emission of volatile metabolites in an artificial tropical rainforest

**DOI:** 10.1038/s41564-023-01507-7

**Published:** 2023-10-06

**Authors:** Linnea K. Hernandez, Giovanni Pugliese, Johannes Ingrisch, Jane Fudyma, Juliana Gil-Loaiza, Elizabeth Carpenter, Esther Singer, Gina Hildebrand, Lingling Shi, David W. Hoyt, Rosalie K. Chu, Jason Toyoda, Jordan E. Krechmer, Megan S. Claflin, Christian Ayala-Ortiz, Viviana Freire-Zapata, Eva Y. Pfannerstill, L. Erik Daber, Kathiravan Meeran, Michaela A. Dippold, Jürgen Kreuzwieser, Jonathan Williams, S. Nemiah Ladd, Christiane Werner, Malak M. Tfaily, Laura K. Meredith

**Affiliations:** 1 Biosphere 2, University of Arizona, Tucson, AZ USA; 2 School of Natural Resources and the Environment, University of Arizona, Tucson, AZ USA; 3 Ecosystem Physiology, Faculty of Environment and Natural Resources, University of Freiburg, Freiburg, Germany; 4 Max Planck Institute for Chemistry, Atmospheric Chemistry Department, Mainz, Germany; 5 Department of Ecology, Universität Innsbruck, Innsbruck, Austria; 6 Department of Environmental Sciences, University of Arizona, Tucson, AZ USA; 7 Joint Genome Institute, Walnut Creek, CA USA; 8 Geo-Biosphere Interactions, Department of Geosciences, University of Tuebingen, Tuebingen, Germany; 9 Environmental Molecular Science Laboratory (EMSL), Earth and Biological Sciences Division, Pacific Northwest National Laboratory, Richland, WA USA; 10 Aerodyne Research, Inc., Billerica, MA USA; 11 Department of Environmental Sciences, University of Basel, Basel, Switzerland; 12 Present Address: Department of Plant Pathology, University of California, Davis, CA USA; 13 Present Address: Bruker Daltonics Inc., Billerica, MA USA; 14 Present Address: Department of Environmental Science, Policy, and Management, University of California, Berkeley, CA USA

**Keywords:** Biogeochemistry, Metagenomics, Microbial ecology, Bacterial genes

Correction to: *Nature Microbiology* 10.1038/s41564-023-01432-9. Published online 31 July 2023.

In the version of this article initially published, there was a mistake in Fig. 2f where ^13^C-diacetyl was plotted instead of ^13^C-enriched diacetyl (^13^C/^12^C + ^13^C). This error led to the erroneous conclusion that there was ^13^C-enriched diacetyl emitted from chambers that received ^13^C1-pyruvate. The corrected version replaces Fig. 2i with ^13^C-enriched diacetyl continuous flux data. Furthermore, since there was no ^13^C-enriched diacetyl emitted from chambers that received ^13^C1-pyruvate, Fig. 2, panel i (as well as panel h), was corrected to show only cumulative ^13^C-fluxes from chambers that received ^13^C2-pyruvate where there were ^13^C-eniched fluxes. Fig. 3, panel a, was corrected to leave only a pink circle under “Diacetyl” indicating ^13^C-enrichment from only chambers that received ^13^C2-pyruvate. Original and corrected Fig. 2f–i and 3a panels are shown as, respectively, Fig. 1 and Fig. 2 below. Finally, in the Results section, the associated text that refers to Fig. 2 in reference to compounds that were ^13^C-enriched from chambers receiving ^13^C1-pyruvate was changed from “Acetic acid and C_4_H_6_O_2_ also showed ^13^C-enriched continuous efflux (Fig. 2e,f) from chambers that received ^13^C1-pyruvate, with cumulative effluxes that increased significantly by factors of 1.9 and 3.5, respectively, during drought (*t*-values = 3.45 and 2.76, respectively; d.f. = 7, *P* < 0.05; LME) (Fig. 2g)” to the following in order to remove mention of diacetyl (C_4_H_6_O_2_): “Acetic acid also showed ^13^C-enriched continuous efflux (Fig. 2e,f) from chambers that received ^13^C1-pyruvate where ^13^C-acetic acid cumulative efflux increased significantly by a factor of 1.9 during drought (*t*-value = 3.45; DF = 7; *P* < 0.05; LME) (Fig. 2g)”. The changes have been made in the HTML and PDF versions of the article.

**Fig. 1:**
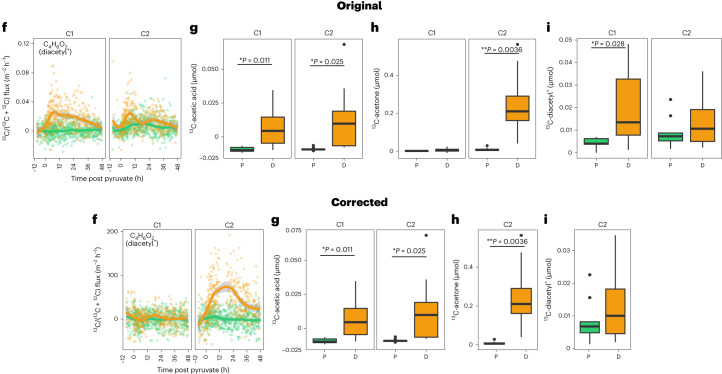
**Original and corrected Fig. 2f–i.**

**Fig. 2:**
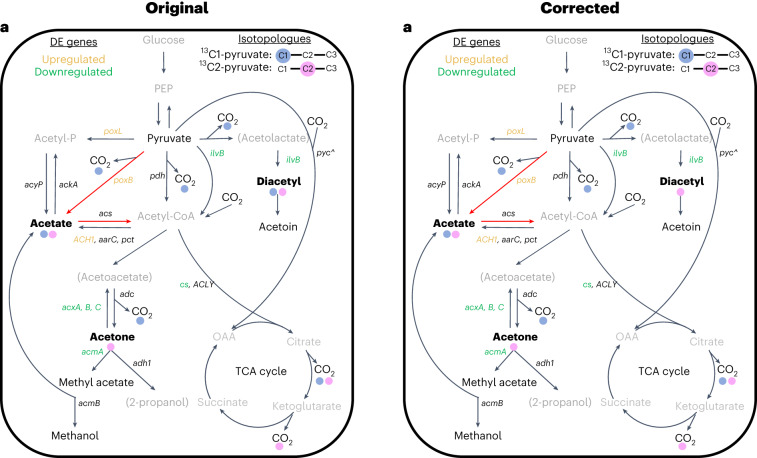
**Original and corrected Fig. 3a.**

