# Supplementary material for: Drought re-routes soil microbial carbon metabolism towards emission of volatile metabolites in an artificial tropical rainforest
Source: Nat Microbiol. 2023 Jul 31;8(8):1480–94. doi: 10.1038/s41564-023-01432-9 (PMC10390333; doi:10.1038/s41564-023-01432-9)
Supplement: Supplementary file 1 — Supplementary Figs.1–8, and Tables 1 and 2. [file 41564_2023_1432_MOESM1_ESM.pdf]

nature microbiology

Article https://doi.org/10.1038/s41564-023-01432-9

Drought re-routes soil microbial carbon metabolism towards emission of volatile metabolites in an artificial tropical rainforest

In the format provided by the authors and unedited

Drought re-routes soil microbial carbon metabolism towards emission of volatile metabolites in an artificial tropical rainforest

Supplementary Information

Extended Data Captions

Extended Data Fig 1. Soil chamber locations and experimental setup. A) Topological map of Biosphere 2 Tropical Rainforest, with locations of Sites 1 - 3. All circles represent collar locations where CO_2 and VOC data were collected and large circles represent a sub selection of sites collars additional samples for metatranscriptomics, metagenomics, and metabolomics were collected. B) Diagram showing soil pyruvate experimental setup with stencil placed inside a soil collar where ^{13}C1- or ^{13}C2-pyruvate was added within each 1 cm x 1 cm square. An automatic soil chamber was placed over the soil collar during the experiment and continuous measurements of ^{12}C and ^{13}C-CO_2 and VOCs were measured.

Extended Data Fig 2. Continuous CO_2 emissions and proportion of C from pyruvate allocated to biosynthesis. A) Total flux of CO_2 during pre-drought and drought conditions. Each point represents a single measurement per soil chamber (~ 64 measurements), of which there were 3 replicates each that were injected with either ^{13}C1- or ^{13}C2-pyruvate for each site (total n =18 each for pre-drought and drought), and lines show the data smoothed with the surrounding shaded area showing +/- SEM. B) Proportion of C from pyruvate allocated to biosynthesis. This was calculated as ^{13}C-$CO_{2\text{-}C1}$/ (^{13}C-$CO_{2\text{-}C1}$ + ^{13}C-$CO_{2\text{-}C2}$) on each set of C1/C2 chambers per site (n = 9 each for pre-drought and drought) with continuous emission data binned to 3 or 6 h intervals, from 0 to 48 h post pyruvate injection. (n= 9 each for pre-drought and drought). Boxes representing Q1 - Q3 with center line indication median, and bars extending to maximum and minimum values, excluding outliers.

Extended Data Fig 3. GC-PTR-TOF data to identify $C_4H_6O_2$. Two nearby locations A) and B) had two peaks with retention times 217 s and 275 s for $C_4H_6O_2$ corresponding to two different compounds. The retention time of 217 s matched the expected retention time for diacetyl, but the retention time of 275 s did not match with any known compounds. Therefore, $C_4H_6O_2$ may represent diacetyl and/or one additional unidentifiable compound, and is referred to as diacetyl$^+$ in the manuscript. The diacetyl peak (retention time of 217 s) is indicated with an arrow.

Extended Data Fig 4. Metatranscriptomic and metagenomic functional and taxonomic diversity shows shifts in active microbial functional profiles and taxonomic diversity, but not in functional potential and total (active and inactive) taxonomic diversity. PCA of (A) metatranscriptomic and (B) metagenomics data. P-values for differences between clusters in PCA was determined by PERMANOVA on Bray-Curtis distance matrices. Taxonomic profiles as inferred from metatranscriptomics data at the (C) kingdom-level for active archaea, bacteria, eukaryota, and viruses, D) phylum-level for bacteria and archaea, and E) phylum-level for fungi. Taxonomic profiles as inferred from metagenomics data at the F) kingdom-level for total archaea, bacteria, eukaryota, and viruses, G) phylum-level for bacteria and archaea, and H) phylum-level for fungi. For taxonomic profiles, arrows indicate direction of changes in relative abundance (up for increased, and down for decreased relative abundance in drought compared to pre-drought conditions). Arrows with no stars means the phylum was present in one condition (pre-drought or drought) and absent in the other condition. For panels A-B, P-values were determined using PERMANOVA on Bray-Curtis distance matrices. For panels C-H, P-values were determined using Linear Mixed Effect Models. *, $p < 0.05$; **, $p < 0.01$; (exact P-values: *P*=0.010 [Proteobacteria], *P*=0.016 [Gemmatimonadetes], *P*=0.0012 [Actinobacteria] (panel B), *P*=0.0050 [Ascomycota] (panel C)). metaT, metatranscriptomics; metaG, metagenomics; B, bacteria; A, archaea; F, fungi

Extended Data Fig 5. Up- or down-regulation of genes encoding for enzymes that cycle ^{13}C-enriched VOCs shift with pyruvate addition. Log_2-fold change (FC) of genes involved in cycling of acetate, acetone, and diacetyl at 6 and 48 h in relation to 0 h to show effect of pyruvate addition on microbial activity during A) pre-drought and B) drought conditions. Log_2-FC values to indicate up-or down-regulation calculated with DESeq2. Production vs.

consumption indicated by black and gray bars, respectively. Significantly up- or down-regulated genes are outlined in color (blue = upregulated, red = downregulated), with exact P-values from DESeq2 analysis shown.); *, $P<0.05$; **, $P<0.01$; ***, $P<0.001$

Extended Data Fig 6. Acetone and acetate cycling gene transcripts. Variance stabilization transformation (VST)-normalized transcript copies of A) acetate- and B) acetone-cycling genes at 0, 6, and 48 h post pyruvate injection. Bars represent Q1 - Q3 with center line indicating median, and bars extending to maximum and minimum values, excluding outliers, across all chambers and sites (n = 9 each for pre-drought and drought).

Extended Data Fig 7. Drought induced a shift in both primary and secondary metabolic composition. A) PCA biplot of samples collected during pre-drought (green; 0 h [n = 5]) or drought (orange; 0 h [n = 5] and 6 h [n = 1]) showing relationships between metabolic profiles identified with NMR which were mostly identified as primary metabolites with relatively low molecular weight. Arrows represent the loadings of individual metabolites. B) PCA biplot of samples collected during pre-drought (green) or drought (orange) showing relationships between profiles of metabolic classes identified with FTICR-MS, capturing mostly high molecular weight metabolites including secondary metabolites. Arrows represent the loadings of metabolic classes driving these patterns. P-values indicate significance of clustering between pre-drought and drought conditions using PERMANOVA on bray-curtis dissimilarity matrix. C) Each stacked bar represents the composition of compound classes identified with FTICR-MS for all samples (n = 18 [6 chambers sampled at 0, 6, and 48 h post pyruvate addition] averaged across pre-drought and drought conditions). Exact P-values are shown as determined by Linear Mixed Effects Models; *, $P<0.05$; **, $P<0.01$; ***, $P<0.001$

Extended Data Fig 8. Modules of co-expressed genes that are associated with pre-drought or drought conditions and ^{13}C-enrichment of VOCs. A) WGCNA cluster dendrogram reveals 9 clusters of co-expressed genes, or modules. The four modules with significant correlations to drought or pre-drought (pink, green, magenta, and brown) are indicated. B) Correlations of the pink, green, magenta, and brown module eigengenes with environmental conditions (pre-drought = 0, drought = 1) and efflux of ^{13}C-enriched (VOC $^{13}C/(^{12}C + ^{13}C)$) acetate and acetone from chambers receiving ^{13}C2-pyruvate (acetate-C2 and acetone-C2, respectively) across three time points (0, 6, and 48 h post pyruvate injection; total n = 27 [3 measurements each for each soil collar receiving ^{13}C2-pyruvate]). Pearson correlation coefficients are shown with FDR-corrected p-values below in parentheses. White boxes indicate no correlation ($P<0.1$). *, $P<0.05$; **, $P<0.01$; ***, $P<0.001$

Extended Data Table 9. Metabolic concentrations (µM) detected using NMR. Samples displayed are a subset of total samples based on the quality of NMR data. Most samples are from 0 h, except for P24 during drought which is from 6 h post pyruvate injection.

**Compound**		**Pre-drought**					**Drought**					
Name	Formula	P15-0hr	P24-0hr	P26-0hr	P34-0hr	P35-0hr	P12-Ctrl	P15-0hr	P24-6hr	P26-0hr	P34-0hr	P34-0hr
2-Oxoisocaproate**P	$C_6H_{10}O_3$	6	5	D	5	D	ND	ND	ND	ND	ND	D
3-Hydroxybutyrate	$C_4H_8O_3$	ND	ND	ND	ND	D	ND	ND	ND	ND	ND	ND
Acetate	$C_2H_4O_2$	22	38	30	55	26	19	35	31	31	24	11
Acetone	C_3H_6O	ND	D	D	ND	ND	D	ND	ND	ND	ND	D
Alanine**P	$C_3H_7NO_2$	24	21	15	20	17	18	9	13	9	7	9
Benzoate	$C_7H_6O_2$	ND	ND	ND	D	ND	ND	ND	ND	ND	ND	ND
Betaine	$C_5H_{11}NO_2$	ND	ND	ND	ND	ND	D	D	ND	ND	ND	ND
Ethanol	C_2H_6O	12	17	21	19	18	28	24	12	22	22	12
Formate***P	CH_2O_2	17	38	40	23	31	ND	D	6	16	D	11
Glutamate	$C_5H_9NO_4$	12	12	8	6	10	11	7	10	10	10	6
Glycerol	$C_3H_8O_3$	6	8	8	6	7	5	6	7	5	8	6
Glycine*P	$C_2H_5NO_2$	12	9	6	8	7	5	D	7	6	D	D
Isoleucine	$C_6H_{13}NO_2$	7	7	D	D	D	D	D	D	D	D	D
Lactate	$C_3H_6O_3$	6	7	D	12	7	D	D	11	D	D	7
Leucine*P	$C_6H_{13}NO_2$	12	11	7	7	6	7	6	6	6	D	D
Methanol	CH_4O	150	375	367	222	326	508	329	203	370	372	322
Phenylalanine*P	$C_9H_{11}NO_2$	D	D	ND	D	ND	ND	ND	ND	ND	ND	ND
Pyroglutamate*P	$C_5H_7NO_3$	22	19	10	16	9	8	5	12	8	D	D
Pyruvate**P	$C_3H_4O_3$	11	13	10	11	10	D	5	9	5	D	7
Succinate	$C_4H_6O_4$	ND	ND	ND	ND	ND	ND	D	D	ND	ND	ND
Threonine	$C_4H_9NO_3$	6	7	D	D	D	D	D	D	D	D	D

Trehalose*[D]	$C_{12}H_{22}O_{11}$	ND	ND	ND	ND	ND	18	D	7	6	13	D
Uracil*[P]	$C_4H_4N_2O_2$	D	5	D	D	D	ND	ND	D	ND	ND	D
Valine*[P]	$C_5H_{11}NO_2$	15	13	8	9	8	8	6	8	6	5	5

ND, not detected (below the level of detection where concentration < 1.9 μM); D, detected (below level of quantification, where concentration 2 - 5 μM)

[P], Compound higher during pre-drought (Linear Fixed Effect Model (DF = 7), where ND and D were given values of 1 and 4 μM respectively, Oxoisocaproate *P*=0.0014, Alanine *P*=0.0074, Formate *P*=4.2E-4, Glycine *P*=0.017, Leucine *P*=0.016, Phenylalanine *P*=0.030, Pyroglutamate *P*=0.018, Pyruvate *P*=0.0011, Uracil *P*=0.022, Valine *P*=0.012)

[D], Compound higher during drought (Trehalose *P*=0.020)

*, $P<0.05$; **, $P<0.01$; ***, $P<0.001$

Extended Data Table 10. Sequence coverage for metagenomics and metatranscriptomics. Read counts are post-filtering (as described in the methods section).

	Metagenomics			Metatranscriptomics		
Sample	Reads	Bases (Gb)	Mapped to assembly (%)	Reads	Bases (Gb)	Mapped to assembly (%)
P11SSC1_190916_c	142713986	21.4	49.1	124166928	18.0	77.9
P11SSC1_190916_f	224647504	33.6	60.0	134601358	19.5	81.2
P11SSC1_190916_i	155786656	23.3	53.4	101082054	14.5	77.6
P11SSC1_191110_c	227806600	34.1	64.9	82931902	12.0	81.1
P11SSC1_191119_f	132381506	19.8	55.9	107176982	15.4	82.3
P11SSC1_191119_i	257335184	38.5	64.8	76710842	11.0	81.1
P12SS_CTRL_191116_c	135568486	20.3	52.9	92715120	13.2	77.0
P12SS_CTRL_191116_f	103105310	15.4	48.0	146171918	20.8	73.4
P12SS_CTRL_191116_i	89432070	13.4	45.8	85117324	12.1	81.9
P15SSC1_190912_c	141457632	21.2	44.5	95565802	13.6	77.1
P15SSC1_190913_f	136093864	20.4	41.0	102381422	14.5	73.1
P15SSC1_190915_i	116566008	17.5	39.7	105544744	15.0	67.5
P15SSC1_191107_f	126857110	19.0	43.0	22699542	3.2	68.0
P15SSC1_191107_i	141678932	21.2	43.5	104936440	14.9	75.1
P15SSCI_191107_c	131734810	19.7	45.6	106270712	15.0	78.9
P24SSC2_190915_c	131640432	19.7	54.6	93137922	13.3	80.2
P24SSC2_190915_f	108754462	16.3	50.0	145088652	20.6	77.5
P24SSC2_190915_i	143103050	21.4	55.0	120662996	17.1	73.7
P24SSC2_191109_c	122604752	18.4	49.7	124732280	17.6	74.2
P24SSC2_191109_f	145461884	21.8	59.2	79879116	11.5	74.6

P24SSC2_191109_i	101194304	15.2	44.3	72649070	10.2	77.6
P26SSC2_190918_c	138246448	20.7	58.3	76395558	10.8	63.8
P26SSC2_190918_f	110179686	16.5	54.9	94868962	13.4	65.5
P26SSC2_190918_i	112067984	16.8	49.2	116842828	16.7	76.7
P26SSC2_191112_c	98449944	14.7	50.9	85137010	12.0	72.2
P26SSC2_191112_f	119958450	18.0	58.9	78330638	10.9	68.4
P26SSC2_191112_i	175881098	26.3	62.4	97683038	13.7	68.9
P34SSC2_190917_c	132462676	19.8	55.5	71554676	10.2	77.8
P34SSC2_190917_f	101846050	15.3	48.6	183218676	25.8	78.6
P34SSC2_190917_i	127521864	19.1	51.5	85630146	12.0	69.7
P34SSC2_191111_c	154773318	23.2	57.8	83204978	12.0	84.2
P34SSC2_191111_f	134298460	20.1	56.9	78791904	10.8	72.9
P34SSC2_191111_i	112046872	16.8	53.5	68499682	9.9	80.1
P35SSC1_190914_c	112270418	16.8	56.0	16633060	2.3	65.2
P35SSC1_190914_f	112035746	16.8	55.5	na	na	na
P35SSC1_190914_i	128627610	19.3	60.0	14394504	2.0	37.3
P35SSC1_191108_c	195950226	29.3	64.4	13623088	1.9	59.7
P35SSC1_191108_f	128984880	19.3	56.8	97460190	13.5	72.1
P35SSC1_191108_i	109718370	16.4	55.1	86907854	12.1	74.3

a, 0 h; f, 6 h; i, 48 h

na, Metatranscriptomics of sample P35SSC1_191108_c were not completed due to sequencing issues
