# Supplementary material for: Drought re-routes soil microbial carbon metabolism towards emission of volatile metabolites in an artificial tropical rainforest
Source: Nat Microbiol. 2023 Jul 31;8(8):1480–94. doi: 10.1038/s41564-023-01432-9 (PMC10390333; doi:10.1038/s41564-023-01432-9)
Supplement: Supplementary file 2 — Reporting Summary [file 41564_2023_1432_MOESM2_ESM.pdf]

nature portfolio

Corresponding author(s): Linnea K. Honeker

Last updated by author(s): Jun 16, 2023

# Reporting Summary

Nature Portfolio wishes to improve the reproducibility of the work that we publish. This form provides structure for consistency and transparency in reporting. For further information on Nature Portfolio policies, see our Editorial Policies and the Editorial Policy Checklist.

## Statistics

For all statistical analyses, confirm that the following items are present in the figure legend, table legend, main text, or Methods section.

| n/a | Confirmed | |
|---|---|---|
| ☐ | ☒ | The exact sample size (*n*) for each experimental group/condition, given as a discrete number and unit of measurement |
| ☐ | ☒ | A statement on whether measurements were taken from distinct samples or whether the same sample was measured repeatedly |
| ☐ | ☒ | The statistical test(s) used AND whether they are one- or two-sided<br>*Only common tests should be described solely by name; describe more complex techniques in the Methods section.* |
| ☐ | ☒ | A description of all covariates tested |
| ☐ | ☒ | A description of any assumptions or corrections, such as tests of normality and adjustment for multiple comparisons |
| ☐ | ☒ | A full description of the statistical parameters including central tendency (e.g. means) or other basic estimates (e.g. regression coefficient) AND variation (e.g. standard deviation) or associated estimates of uncertainty (e.g. confidence intervals) |
| ☐ | ☒ | For null hypothesis testing, the test statistic (e.g. *F*, *t*, *r*) with confidence intervals, effect sizes, degrees of freedom and *P* value noted<br>*Give P values as exact values whenever suitable.* |
| ☒ | ☐ | For Bayesian analysis, information on the choice of priors and Markov chain Monte Carlo settings |
| ☒ | ☐ | For hierarchical and complex designs, identification of the appropriate level for tests and full reporting of outcomes |
| ☐ | ☒ | Estimates of effect sizes (e.g. Cohen's *d*, Pearson's *r*), indicating how they were calculated |

*Our web collection on statistics for biologists contains articles on many of the points above.*

## Software and code

Policy information about availability of computer code

| | |
|---|---|
| Data collection | no software used |
| Data analysis | R v. 4.0.2, (packages: NLME v. 3.1, WGCNA v. 1.7, DESeq2 v. 1.30.1, ggplot2 v. 3.4.1, pls v. 2.8, plsVarSel v. 0.9.10, ClusterProfiler v. 3.18.1, FLUX v. 0.3), BBtools (BBDuk v. 38.94, BBMap v. 38.44), metaSPAdes v. 3.15.12, INFERNAL v. 1.1.3, Prodigal v. 2.6.3, GeneMarkS-2 v. 1.07, tRNAscan-SE v. 2.0.6, BrukerDaltonik v. 4.2, PTRwid, MetaboDirect v. 0.2.7, Chenomx NMR suite 9.2, MestReNova, For)mularity Complete R code for all data analysis to produce figures is included in the following GitHub repository (https://github.com/linneakh/SoilPyruvate) |

For manuscripts utilizing custom algorithms or software that are central to the research but not yet described in published literature, software must be made available to editors and reviewers. We strongly encourage code deposition in a community repository (e.g. GitHub). See the Nature Portfolio guidelines for submitting code & software for further information.

## Data

Policy information about availability of data

All manuscripts must include a data availability statement. This statement should provide the following information, where applicable:

- Accession codes, unique identifiers, or web links for publicly available datasets
- A description of any restrictions on data availability
- For clinical datasets or third party data, please ensure that the statement adheres to our policy

The metatranscriptomics and metagenomics sequence data are publicly available through Genbank SRA under the following BioProject ID's: PRJNA980752-

PRJNA980834. FTICR, NMR, VOC, and CO2 data are deposited to FigShare (OOI: 10.6084/m9.figshare.20334537), and individual raw .xml files for the FTICR data are included as Supplementary Data. The KEGG database can be accessed at the following website: https://www.genome.jp/kegg/pathway.html

## Human research participants

Policy information about studies involving human research participants and Sex and Gender in Research.

| | |
|---|---|
| Reporting on sex and gender | This information has not been collected and is not applicable to this study |
| Population characteristics | This information has not been collected and is not applicable to this study |
| Recruitment | This information has not been collected and is not applicable to this study |
| Ethics oversight | This information has not been collected and is not applicable to this study |

Note that full information on the approval of the study protocol must also be provided in the manuscript.

# Field-specific reporting

Please select the one below that is the best fit for your research. If you are not sure, read the appropriate sections before making your selection.

☐ Life sciences ☐ Behavioural & social sciences ☒ Ecological, evolutionary & environmental sciences

For a reference copy of the document with all sections, see nature.com/documents/nr-reporting-summary-flat.pdf

# Ecological, evolutionary & environmental sciences study design

All studies must disclose on these points even when the disclosure is negative.

| | |
|---|---|
| Study description | Our study examined the impact of drought on microbial carbon cycling using position-specific 13C-pyruvate labeling, to determine how carbon is allocated to VOCs and CO2, combined with multi-omics. For VOC and CO2 data, continuous measurements were made from 0 to 48 hrs post 13C-pyruvate injection across 6 replicates each within 3 sites of the Biosphere 2 tropical rainforest (n = 18) during both pre-drought and drought conditions (total n = 36). For metabolomics, metatranscriptomics, and metagenomics, data are from discrete time points when soil was collected (0, 6, and 48 hr post pyruvate injection). Due to our limited number of samples that could be analyzed at EMSL, we sent a subset of the sites for analysis of 2 each from sites 1, 2 and 3 (for 3 time points, n= 18) for pre-drought and drought (total n=36). |
| Research sample | The sample unit originated from a region of soil enclosed by a soil gas chamber with a diameter of 20 cm. For gas measurements, the soil chamber was closed and continuous CO2 and VOC measurements were made (every 30 min for first ~ 8 hr, then every 50 min until 48 hr). For soil samples, a 2.2 cm diameter metal ring was pushed into soil to a depth of 2 cm. |
| Sampling strategy | No calculations were made to determine a priori how many samples should be collected. A replicate size of 6 per site was chosen based on feasibility of instrumentation and connection to gas analyzers. A replicate size of 2 per site was chosen for multi-omics based on the limited number of samples we could have analyzed at JGI and EMSL with the FICUS grant. We selected 2 per site rather than 3 each for 2 sites because we wanted to capture the heterogeneity across the tropical rainforest biome and focus on the pre-drought vs drought conditions rather then comparisons between sites. |
| Data collection | CO2 and VOC data, including isotopic composition, were collected by Johannes Ingrisch, Eva Pfannerstill, Jordan Krechmer, and Megan Claflin using multiplexed Licor soil flux system (Licor 8100, Li-8150, and Lic 8100-104) coupled to a Picarro G2201-i analyzer and proton-transfer-reaction time-of-flight spectrometer (PTR-TOF 8100). Metatranscriptomic and metagenomic data were collected by the joint genome institute (JGI) on an Illumina NovaSeq. Metabolomics data were collected at EMSL (PNNL) on an fourier transform ion-coupled resonance (FTICR) mass spectrometer for larger metabolites, and a nuclear magnetic resonance (NMR) spectrometer for small metabolites. Soil moisture data were collected by various researchers on a handheld probe coupled to a LabQuest viewer. |
| Timing and spatial scale | 13C-pyruvate injection experiments and sample collections were performed between September 12 - 15 and November 17-19 for pre-drought and drought conditions, respectively. Per sampling location/chamber, gas measurements were made for 48 hours post 13C-pyruvate injection, and for multi-omics, samples were collected and analyzed from 0, 6, and 48 hr time points post pyruvate injection. |
| Data exclusions | No data were excluded |
| Reproducibility | There were no attempts to reproduce the experiment due to the immense time, costs, and manpower (international research team) required to conduct the drought experiment. We tried to include sufficient replicates to ensure confidence in our experimental results. |
| Randomization | Samples were not randomized into groups, due to the nature of field collection from distinct locations and time points. |

| | |
|---|---|
| Blinding | Gas data acquisition could not be blinded due to the nature of the study. Genomic and metabolomic data acquisition was blinded because the individuals collected the data were not familiar with the project or sample IDs. |

Did the study involve field work? ☒ Yes ☐ No

## Field work, collection and transport

| | |
|---|---|
| Field conditions | During ambient (pre-drought) conditions, rainfall events were simulated by spraying 15,000 L of irrigation water from the top of the B2 TRF at a frequency of 3 days a week. The last rainfall event before the drought was on October 7, 2019, and the first post-drought rain-fall was on December 12, 2019. During the drought, ambient temperatures were maintained in the lowland region between 20 to 26.7 °C. |
| Location | Tropical rainforest biome at Biosphere 2 in Oracle, AZ. |
| Access & import/export | Our study site was not part of a natural ecosystem, so we did not have to adhere to any specific guidelines for minimizing our disturbance to the site. The Biosphere 2 is a 1.27 ha steel and glass-enclosed building that is highly accessible for research. Samples were collected, stored in coolers with dry ice and then moved to refrigerators/freezers on site. From there, samples were transported to the University of Arizona, about 40 miles away. |
| Disturbance | Disturbance was minimal with soil samples collected only to a depth of 2 cm at 18 sites. |

# Reporting for specific materials, systems and methods

We require information from authors about some types of materials, experimental systems and methods used in many studies. Here, indicate whether each material, system or method listed is relevant to your study. If you are not sure if a list item applies to your research, read the appropriate section before selecting a response.

### Materials & experimental systems

| n/a | Involved in the study | |
|---|---|---|
| ☒ | ☐ | Antibodies |
| ☒ | ☐ | Eukaryotic cell lines |
| ☒ | ☐ | Palaeontology and archaeology |
| ☒ | ☐ | Animals and other organisms |
| ☒ | ☐ | Clinical data |
| ☒ | ☐ | Dual use research of concern |

### Methods

| n/a | Involved in the study | |
|---|---|---|
| ☒ | ☐ | ChIP-seq |
| ☒ | ☐ | Flow cytometry |
| ☒ | ☐ | MRI-based neuroimaging |
